# Novel Proteome Extraction Method Illustrates a Conserved Immunological Signature of MSI-H Colorectal Tumors

**DOI:** 10.1074/mcp.RA120.002152

**Published:** 2020-11-25

**Authors:** Elez D. Vainer, Juliane Kania-Almog, Ghadeer Zatara, Yishai Levin, Gilad W. Vainer

**Affiliations:** 1 Department of Gastroenterology, Hadassah—Hebrew University Medical Center, Jerusalem, Israel; 2 Tel Aviv Sourasky Medical Center, Sackler School of Medicine, Tel Aviv Sourasky Medical Center, Tel Aviv, Israel; 3 Department of Pathology, Hadassah—Hebrew University Medical Center, Jerusalem, Israel; 4 De Botton Institute for Protein Profiling, The Nancy and Stephen Grand Israel National Center for Personalized Medicine, Weizmann Institute of Science, Rehovot, Israel

**Keywords:** Clinical proteomics, colorectal cancer, immunohistochemistry, immunology, molecular biology, label-free quantification, inflammatory response

## Abstract

Using a simple, environment friendly proteome extraction (TOP), we were able to optimize the analysis of clinical samples. Using our TOP method we analyzed a clinical cohort of microsatellite stable (MSS) and unstable (MSI-H) colorectal carcinoma (CRC). We identified a tumor cell specific, STAT1-centered, immune signature expressed by the MSI-H tumor cells. We then showed that long, but not short, exposure to Interferon-γ induces a similar signature *in vitro*. We identified 10 different temporal protein expression patterns, classifying the Interferon-γ protein temporal regulation in CRC. Our data sheds light on the changes that tumor cells undergo under long-term immunological pressure *in vivo*, the importance of STAT proteins in specific biological scenarios. The data generated could help find novel clinical biomarkers and therapeutic approaches.

## Teaser

The TOP method is a simple, robust, environment friendly proteome extraction method, with decreased fixation time bias. Using the TOP method, we analyzed by LC\MS-MS a clinical cohort of microsatellite stable (MSS) and unstable (MSI-H) colorectal carcinoma (CRC). An MSI-H specific, STAT-1 centric, immunological signature was identified. In-vitro experiments connected this signature to long, but not short exposure, to Interferon-g. Our data provides in-depth view of the MSI-H immunobiology and suggests that the roles of STAT proteins are context dependent.

## Graphical Abstract

**Figure:**
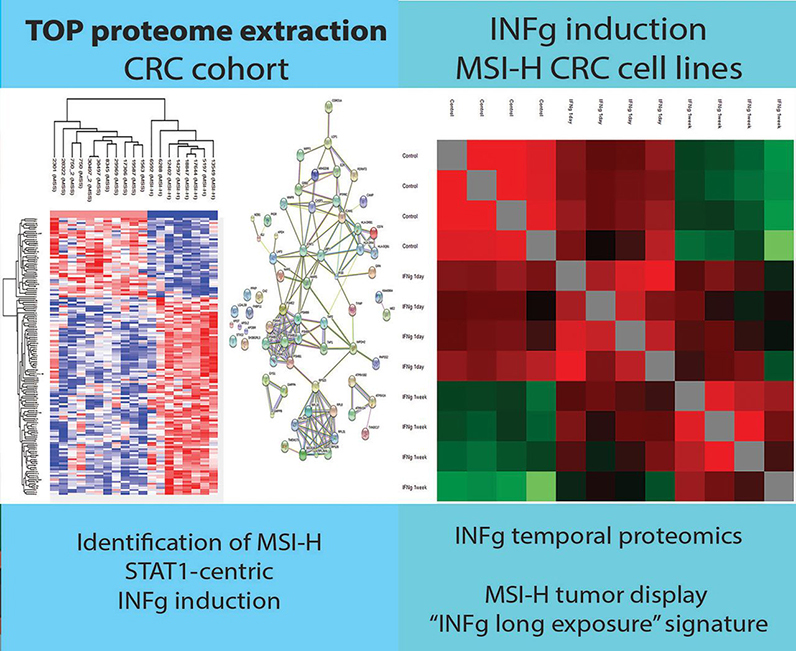

• TOP: robust, bio-friendly FFPE proteome extraction method with less fixation bias.
• Proteome of MSI-H colorectal cancer identifies immunobiology key elements.
• MSI-H tumor displays an “INFg-STAT1 centric signature”.
• Long-term IFNg induction In-vitro mimicks MSI-H signature.

Formalin fixation is the standard in clinical routine for more than 100 years. Indeed, one of the major breakthroughs in molecular pathology was the effective extraction of DNA and then RNA from formalin-fixed and paraffin-embedded (FFPE) clinical tissues. Although some progress was made to establish a robust protein extraction method (1, 2), the issue was found to be complex (3). One of the major inherent problems is the huge variability of tissue fixation time in the clinics. In the clinics, it is adequate to fix tissues for 6 h to 72 h (4). This creates a huge bias for traditional proteomics pipelines, as protein recovery seems to decrease with prolongation of fixation time, with yields dropping by ∼20% for every 24 h in formalin (5). Thus, a robust, efficient, fixation-time impervious protein extraction method will help to propel clinical molecular proteomics forward.

Colorectal cancer (CRC) is a common cancer and one of the leading causes of cancer related death (6). In CRC genomic instability seems to be a key developmental factor. Two main forms of genomic instability govern the process: chromosomal instability and microsatellite instability (MSI) (7). Most CRC cases are characterized by chromosomal instability with microsatellite stability (MSS). However, ∼5–15% of CRC are microsatellite instable (MSI-H) (7, 8).

The MSI-H tumors, also known as mismatch repair deficient (MMRd), develop because of a deficiency of the MMR proteins. The MMRd might result from epigenetic silencing (“sporadic”) or by constitutional mutations (“genetic”) (8). In addition, the MSI-H tumors are typically characterized by a high rate of single-nucleotide substitutions (9). On average, these tumors display a high mutation rate of 12-40 mutations/Mb (7). As a result, MSI-H tumors can produce abnormal proteins that might act as neo-antigens and trigger an effective immune response against the tumor (10). There is an increasing body of evidence that shows a unique and complex interplay between the MSI-H tumor cells and the immune microenvironment. Although much is unknown, it has been shown that MSI-H tumors show an immune active tumor microenvironment with high levels of several immune checkpoint proteins (11).

Recently, the Food and Drug Administration (FDA) granted accelerated approval to Pembrolizumab (Keytruda) for the treatment of MSI-H (MMRD) solid tumors arising from different organs, thus granting the first histology agnostic treatment approval. This was based on data collected from several KEYNOTE clinical trials that enrolled MSI-H cancer patients irrespectively of the tumor origin. Thus, clinically and biologically MSI-H CRC might be considered as enriched for PD-1 immune checkpoint inhibition responsive, whereas MSS CRC as unresponsive.

The JAK-STAT axis is one of the most important pathways in cancer (12). Specifically, the pleiotropic transcription factors STAT1/3 appear to have a complex and conflicting role in CRC (13). In general, STAT1 is assumed to be a tumor suppressor (13). However, although it was shown to correlate with a better prognosis (14, 15), it was also claimed to be associated with shorter survival and poor outcome, particularly of the microsatellite instable subtype (16). Moreover, some support the concept of STAT3 as an oncogene, whereas others as a tumor suppressor (13). Contradictory reports were published, with some who describe the oncogenic effects of STAT3 [17, 18, 19, 20], whereas others the positive outcome (15, 21).

Here we investigated the proteomes of MSI-H *versus* MSS CRC using a novel clinical proteome pipeline. We have found that the proteins overexpressed by the MSI-H, but not the MSS tumors, displayed a dense protein network. The network was highly enriched for immunological processes, and specifically for antigen processing and presentation. Interestingly, only STAT1 was overexpressed by the MSI-H tumors, and only STAT1 showed a strong connection to immune-related processes. Furthermore, we managed to reproduce some of these changes *in vitro* by long-, but not short-, exposure to Interferon-γ. We continued and investigated the temporal proteomics of Interferon-γ exposure. Our data sheds new light on the basic immunobiology of MSI-H CRC.

## MATERIALS AND METHODS

##### Xylene-Mediated (Traditional) Proteome Extraction

The traditional protein extraction was done according to the Mann's laboratory manuscripts (22, 23). Xylene-mediated deparaffinization was accomplished by a 1 ml xylene incubation at 37˚C for one hour. After vortexing and 5 mins centrifugation at 20,000 × *g*, xylene was removed. A second incubation with fresh xylene for 30 min at 37˚C followed, again ensued by vortexing, centrifugation and removal of xylene. Then, a wash with 1 ml of absolute ethanol was done for 5 min at room temperature. After steps of vortexing and centrifugation like above, ethanol was removed. Ethanol wash was repeated one more time. Ethanol was discarded and remaining pellets were air-dried for 30 min upside down.

For protein recovery, pellets were resuspended in 0.1 M Tris pH 7.5. Subsequently, samples were sonicated at level 5 for 3 min on ice (Fisher Scientific, Massachusetts). After addition of SDS (SDS) to a concentration of 4% and a total buffer volume of 150 μl, samples were heated up to 95˚C for one hour in a dry bath (Elite EL-02, MS major science, New York). Following 10 min centrifugation at 20,000 × *g*, supernatants were transferred to new tubes and stored at −70˚C. All reagents are from Bio-Lab, unless stated otherwise.

##### TOP Proteome Extraction

Deparaffinization was conducted using mineral oil (Ventana, arizona). Sections were incubated in 1 ml of mineral oil at 90˚C for 30 min with agitation. Then, 130 μl of 0.1 M Tris pH 7.5 buffer was added. The tubes were rigorously mixed and left for 5 min to start tissue rehydration, then centrifuged at 20,000 × *g* for 5 min. Mineral oil (the upper phase) was removed carefully. Like the xylene-mediated deparaffinization, the samples were sonicated and SDS was added. Samples were heated up for 5 min at 121˚C (∼15 psi) in an autoclave (Wet cycle; Tuttnauer, Jerusalem, Israel). Subsequent centrifugation, transfer of supernatants and storage were performed as stated above.

##### Clinical Cohort and Proteome Extraction

A colorectal carcinoma tumor cohort was collected under ethical approval of the Tel Aviv Sourasky Medical Center ethics committee (supplemental Table S1). 19 surgery samples from 8 MSI-H and 9 MSS different patients (including two biological duplicates from 2 MSS patients). Blocks were sliced to consecutive 8 μm sections with a microtome (RM2265, Leica Biosystems, Wetzlar, Germany). Macro-dissection was done to ensure a minimum of 50% tumor cells in the sample. The macro-dissected material was moved into 1.5 ml. Eppendorf tubes. The proteome was extracted using TOP extraction (see above).

##### Proteome of Cell Line

As in the western experiments, 0.5 × 10^6^ DLD1 cells were seeded in 6-well plate for a week. Then, Interferon-γ was immediately added for 1-week group, or after 6 days it was added for the last day in the 1-day group. Control cells cultured without Interferon-γ. The experiment was done in quadruplicates. At the end of experiment, cells were washed with 0.9% NaCl and lysed with 100 mm Tris pH7.5, 4% SDS. The protein extracts were kept at −70 °C until analysis.

##### Sample Preparation for Label-Free Mass Spectrometry

Samples were subjected to in-solution tryptic digestion using a modified filter aided sample preparation protocol (FASP) (24). All chemicals were sourced from Sigma Aldrich unless stated otherwise. Sodium dodecyl sulfate buffer (SDT) included: 4%(w/v) SDS, 100 mMTris/HCl pH 8, 0.1 M DTT. Urea buffer (UB): 8 M urea in 0.1 M Tris/HCl pH 8.0. 15 μl of the sample, in SDT buffer, were mixed with 200 μl UB, loaded onto 30 kDa molecular weight cutoff filters and centrifuged. 200 μl of UB were added to the filter unit and centrifuged again. Proteins were alkylated using 10 mm iodoacetamide (Sigma) for 45 min, at room temperature in the dark. Filters were washed twice with 50 mm ammonium bicarbonate followed by trypsin digestion. Trypsin was added and samples were incubated at 37 °C overnight. At the next day, trypsin was added again for 4 h. Digested proteins were then spun down, acidified, desalted, and then stored in −80 °C until analysis.

##### NanoLC MS ESI MS/MS Analysis

ULC/MS grade solvents were used for all chromatographic steps. Each sample was loaded using split-less nano-ultra performance liquid chromatography (Waters). The mobile phase was: (a) H_2_O + 0.1% formic acid and (b) acetonitrile + 0.1% formic acid. Desalting of the samples was performed online using a reversed-phase C18 trapping column (180 μm internal diameter, 20 mm length, 5 μm particle size; Waters). The peptides were then separated using a HSS T3 nano-column (75 μm internal diameter, 250 mm length, 1.8 μm particle size; Waters). Peptides were eluted from the column into the mass spectrometer using the following gradient: 4% to 35% B in 150 min, 35% to 90%B in 5 min, maintained at 95% for 5 min and then returned to initial conditions.

The nanoUPLC was coupled online through a nanoESI emitter (10 μm tip; New Objective, Woburn, MA, USA) to a quadrupole orbitrap mass spectrometer (Q Exactive Plus, Thermo Scientific) using a FlexIon nano spray apparatus (Thermo Scientific).

Data were acquired in data dependent acquisition (DDA) mode, using a Top20 method. MS1 resolution was set to 70,000 (at 400 *m*/*z*), automatic gain control (AGC) to 3e^6^ and maximum injection time was set to 20 msec. MS1 isolation window was set to 1.6 mass units. MS2 resolution was set to 17,500, AGC to 1e^6^ and maximum injection time of 60 ms.

##### Protein Identification and Quantification

Raw data were processed using MaxQuant (MQ) version 1.6.5.0 (25) and the embedded Andromeda search engine (26). Data were searched against the human sequences in Swissprot human proteome version 2017_01 (20299 entries). Precursor mass and fragment mass were searched with mass tolerance of 4.5 and 20 ppm, respectively. The search included variable modifications of oxidation (methionine) and protein N-terminal acetylation, and fixed modification of carbamidomethyl (cysteine). Enzyme specificity was set to trypsin, a maximum of two miscleavages were allowed, and a minimal peptide length was set to 7 amino acids. The false discovery rate (FDR) for peptide and protein identifications was 0.01. For the conventional *versus* TOP proteome extraction the defaults were kept, except: deamidation (NQ) was added as variable modification, and match between runs was not enabled.

The bioinformatics was performed on Perseus suite (version 1.6.2.3). The data were filtered for reverse, contaminants and identified by site. Then the data were filtered such that a protein had to have none-zero LFQ intensity in all 19 samples with 3 or more peptides, unless stated otherwise. Gene Ontology annotation performed using STRING site version 10.5.

##### Immunohistochemistry

All immunohistochemistry was done on the Benchmark XT automated machine (Ventana), using FDA-approved reagents (UltraView kit) with amplification (Ventana). The antigen retrieval solution was CC1 (Ventana). All primary antibodies were obtained from Abcam, Cambridge, United Kingdom (Anti-MHC Class II antibody (ab157210), Anti-HLA-DQB1 antibody (ab183898), Anti-CD74 antibody (ab22603), Anti-HLA-DPB1 antibody (ab193392).

##### Cells

DLD1 and RKO cells were cultured in DMEM (biological industries) supplemented with 10% FCS, 2 mm Glutamine and antibiotics. Interferon-γ (Peprotech) was added to the medium as indicated.

##### Western Blotting

On day of experiment, 0.5 × 10^6^ DLD1 and RKO cells were seeded on 6-well plates, treated with Interferon-γ and incubated at 37 °C in humidified atmosphere containing 5% CO_2_. For concentration scale experiments, cells were incubated with different concentrations of Interferon-γ (1, 5, 10, 20 and 40 ng/ml). For time scale experiments, cells were incubated with 10 ng/ml Interferon -γ for 15 min, 1 h, 6 h and 24 h. At the end of experiment, cells were briefly washed with 0.9% NaCl and lysed with lysis buffer (100 mm Tris pH7.5, 5% SDS). For Western blot analysis, cells were sonicated and centrifuged at 12,000 × *g* for 10 min. Protein extract was subjected to 10% SDS/PAGE and transferred to nitrocellulose membranes. After blocking in 0.15% gelatin (Sigma), blots were incubated with primary antibodies (Cell Signaling) for: Total STAT1 (9172), and Total STAT3 (8019) for overnight, at 4 °C. Blots were washed in TBS − 0.2% Tween20 (TBS-T) three times, and incubated for one hour at room temperature with HRP-conjugated secondary antibody diluted in 0.15% gelatin. Bands were visualized by ECL, and detected using C600 camera (Azure, Dublin, CA).

To control protein loading amounts, all gels were prepared with 2,2,2-Trichloroethanol (TCE; Sigma). At the end of electrophoresis, gels were automatically imaged (TCE program, C600 camera; Azure).

##### Experimental Design and Statistical Rationale

For the LC–MS/MS analysis comparison of TOP method to the conventional proteome extraction we used three independent biological replicate experiments. The tissue after 2 days fixation served as a control.

For the LC–MS/MS analysis comparison of the MSS and MSI-H proteome, we used 19 independent biological extractions. The MSS group served as a control.

For the LC–MS/MS analysis of DLD1 exposure to Interferon-γ we used four independent biological replicate experiments. The DLD1 cells without Interferon-γ exposure served as a control.

The bioinformatics was done on Perseus suite (version 1.6.2.3). The significantly enriched proteins were found (two-sample *t* test with a permutation-based FDR method) and further selected using an adjusted *p* value <0.05. Log2-transformed individual values or triplicate means were z-score-normalized prior to hierarchical clustering. Gene Ontology annotation performed using STRING site version 10.5, and the statistical values by the site have been reported.

## RESULTS

##### Optimization of FFPE Proteome Extraction

Formalin fixation times vary considerably in routine clinical setting (4). One of the consequences is the deleterious impact of extended fixation time on the amount of protein extracted from clinical FFPE samples, which influences the proteomic coverage achievable by MS analysis (5, 27). Therefore, we aimed to minimize the fixation length effects.

To enhance the extraction, we changed several steps in the proteome extraction: (1) Deparaffinization using mineral oil excluded toxic organic solvents and multiple washes. (2) Elevated temperature and pressure (121˚c, 15 psi) to aid the protein extraction process. We named our proteome extraction TOP (Temperature-Oil-Pressure) method.

We compared the conventional and the TOP proteome extraction using mass spectrography. We used a set of matched tissues fixed in formalin for 2 or 8 days, representing clinically adequate or over-fixation, respectively. This created four groups named conventional-2d, conventional-8d, TOP-2d and TOP-8d.

**Fig. 1:**
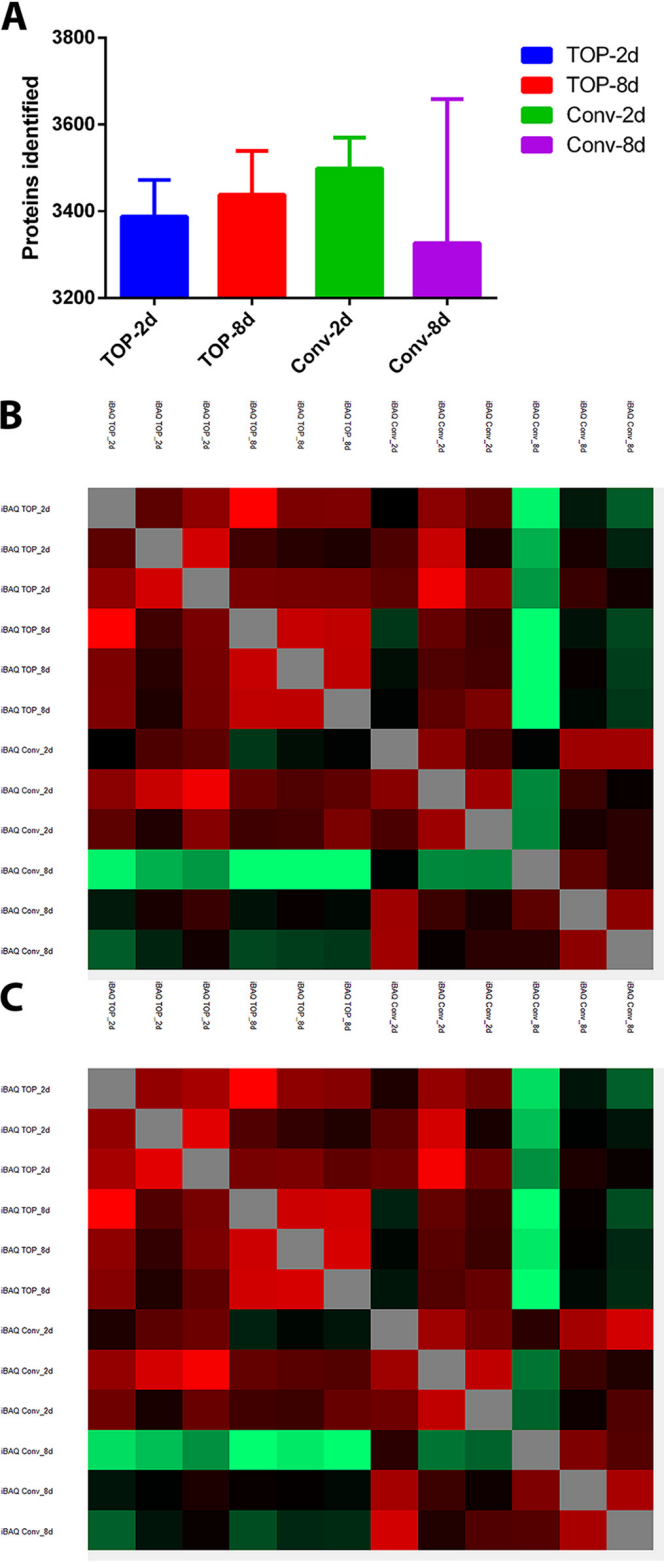
**TOP method is more resistant to formalin fixation related biases than the traditional proteomics extraction.***A*, Head-to-head comparison of the number of proteins identified in all experimental arms. *B*, Heat-map of Pearson's correlation score using 3988 proteins identified. *C*, Heat-map of Pearson's correlation score of 2350 proteins identified in all 12 samples.

First, MQ iBAQ identified 5240 protein groups, but filtering for site, reverse, contaminants, and protein with at least 3 peptides left us with 3988 proteins. We identified a comparable number of proteins in the four groups (Fig 1*A*). Then, we studied the Pearson's correlation (all 3988 proteins identified). The analysis pointed out that the conventional-8 days' group showed less correlation to the other groups (Fig. 1*B*).

Because the Conventional-8day groups showed slightly decreased amounts of identified proteins, we wanted to determine if this was the cause of the decreased correlation. Thus, we repeated the same analysis but with the proteins identified in all 12 samples (2350 proteins). As before, the proteomes of the Conventional-8day group showed the lowest correlation. Importantly, the proteomes of this group showed a high correlation to each other, suggesting that there was a consistent bias in this group (Fig. 1*C*).

##### Unsupervised Clustering of CRC TOP Samples Follows CRC Biology

We decided to test our improved TOP proteome extraction method on clinical formalin-fixed paraffin-embedded samples. We focused on microsatellite stable (MSS) and unstable (MSI-H) colorectal carcinoma. The colorectal TCGA study clearly showed that the MSI status has a profound impact on the tumor biology (7), and the FDA granted the first tissue agnostic approval for immunotherapy treatment in MSI-H patients (28, 29). Surprisingly, the TCGA proteomics effort identified five proteomic subtypes in the TCGA cohort, with only weak association to MSS/MSI-H groups (30, 31).

To investigate this point, the proteomes of 19 samples were extracted by the TOP method, eight of which were MSI-H (42%). We identified 4350 proteins but continued with only 1664 proteins that were identified in all 19 samples. Our clinical cohort showed a high Pearson's correlation between the samples (supplemental Table S1 and supplemental Fig. S1), with Pearson's correlation of ∼0.98 for biological replicates. This supports the notion that the TOP proteome extraction produces highly consistent proteome results from clinical FFPE samples.

**Fig. 2:**
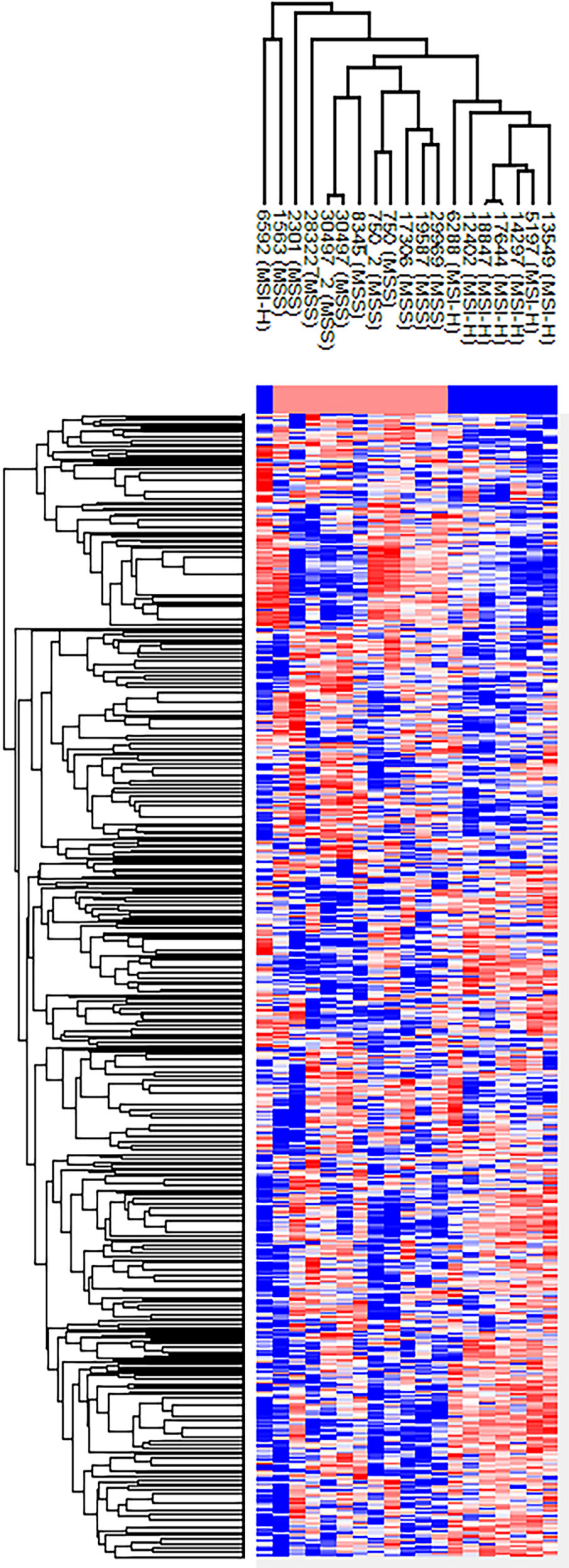
**Unsupervised hierarchical clustering (Euclidean distance based).** Two main clusters seen correspond to the MSI-H and MSS groups. The two biological replicates (samples 750 and 30497) cluster together as expected and serve as a clustering internal control. These reflect the high Pearson correlation scores observed between the two biological replicates of cases 750 and 30497 (0.978 and 0.982, respectively; See supplemental Fig. S1). The row clustering tree shows two main clusters, as expected.

Importantly, in agreement with the original TCGA publication (7), an unsupervised hierarchical clustering of the cohort shows two major clusters (Fig. 2). One of the main clusters contained the MSI-H samples (7 out of 8), whereas the other contained the MSS samples (8 out of 11). We noted no MSI-H in the MSS cluster, and vice versa. Additionally, the two biological duplicates (different areas of the same tumor) cluster tightly together (Samples 750 and 30497; Fig. 2), highlighting again the reproducibility of the pipeline.

These results suggest that when optimized, clinical modern proteomics can produce consistent, high quality data and that the tumor proteome reflects the MSI status.

##### MSI-H Tumors Overexpress STAT1 and Numerous Immune-Related Proteins

**Fig. 3:**
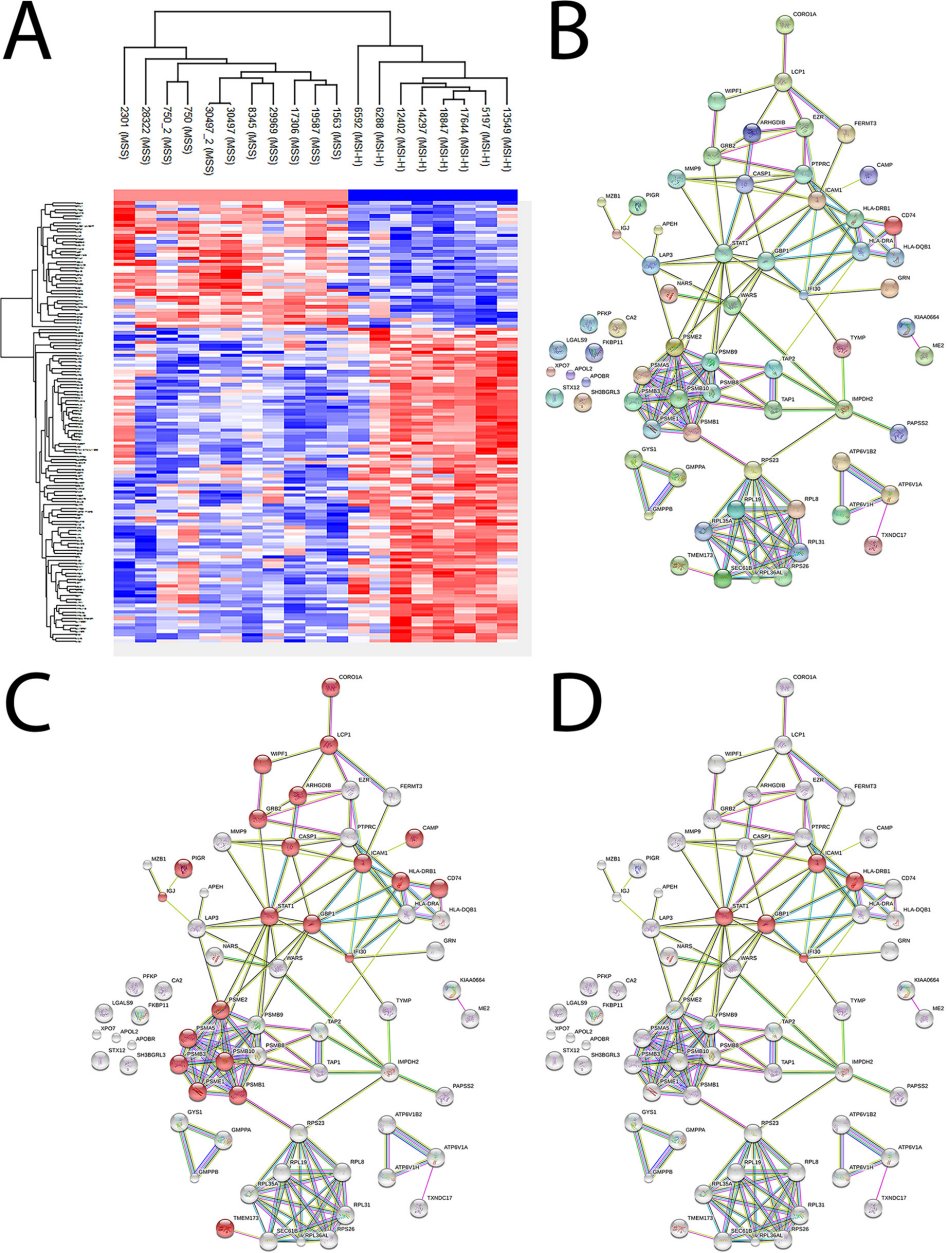
**67 proteins overexpressed in MSI-H tumors (FDR < 0.05, S = 0.1).***A*, Supervised hierarchical clustering showing two clusters formed using the proteins found to be differentially expressed (Euclidean distance based). *B*, Graphical representation of the 67 proteins found to be overexpressed in MSI-H. STRING analysis showed that the proteins have a statistically significant increase in interactions, with 144 edges seen (expected number was 43). *C*, 22 proteins are related to immune response (GO6955; FDR 5.12e^−08^). *D*, 5 proteins are related to interferon-gamma-mediated signaling pathway (GO60333; FDR 3.87e^−04^). The proteins involved in the process are highlighted in red.

We investigated the proteins that showed significant differential amounts between the MSI-H and MSS groups. Using permutation-based false detection rate (FDR *p*-Value < 0.05, S = 0.1), we found 67 and 35 proteins to be overexpressed in the MSI-H or MSS group, respectively (Fig. 3*A*; supplemental Table S2).

Using the STRING v11 database, we investigated the connections of the proteins (32). We found no statistically significant enrichment in the 35 proteins overexpressed by the MSS tumors. In sharp contrast, the STRING analysis found a highly significant connection enrichment in the MSI-H overexpressed proteins (Fig. 3*B*; *p*-Value < 10^−16^). Furthermore, ∼33% of the proteins (22 out of 65) were found to be linked to immune response processes (Fig. 3*C*), and specifically to the Interferon-γ-mediated signaling pathway (Fig. 3*D*) and antigen processing and presentation (FDR 7.9e^−8^). In agreement, the three top ranked KEGG enrichment pathways were the proteasome (8 proteins), the phagosome (10 proteins) as well as antigen processing and presentation (8 proteins) (FDR *p*-Value 3.2e^−10^, 4.06e^−9^ and 4.06e^−9^, respectively; full GO and KEGG lists are in supplemental Table S2).

Interestingly, the list included signal transducers and activators of transcription 1 (STAT1), a key transcription factor in many immunological processes, including Interferon-γ signaling (see below) (33, 34, 35). It is important to remember that this correlation is to the total amount of STAT1 and not to the phospho-form (active) of STAT1 protein.

Taken together, we show that the proteins overexpressed by MSI-H tumors are highly enriched and related to the immune system. Specifically, we found a significant elevation of STAT1 levels, and proteins related to MHC-II and antigen presentation.

##### MSI-H Colorectal Tumor Cells, and Not the Stroma Cell, Aberrantly Express MHC-II Receptors

Our proteomics data lacks morphological annotation, and it was not clear which component in the tissue overexpresses these proteins, the tumor or the nonmalignant stromal component that includes many immune cells (36, 37, 38).

**Fig. 4:**
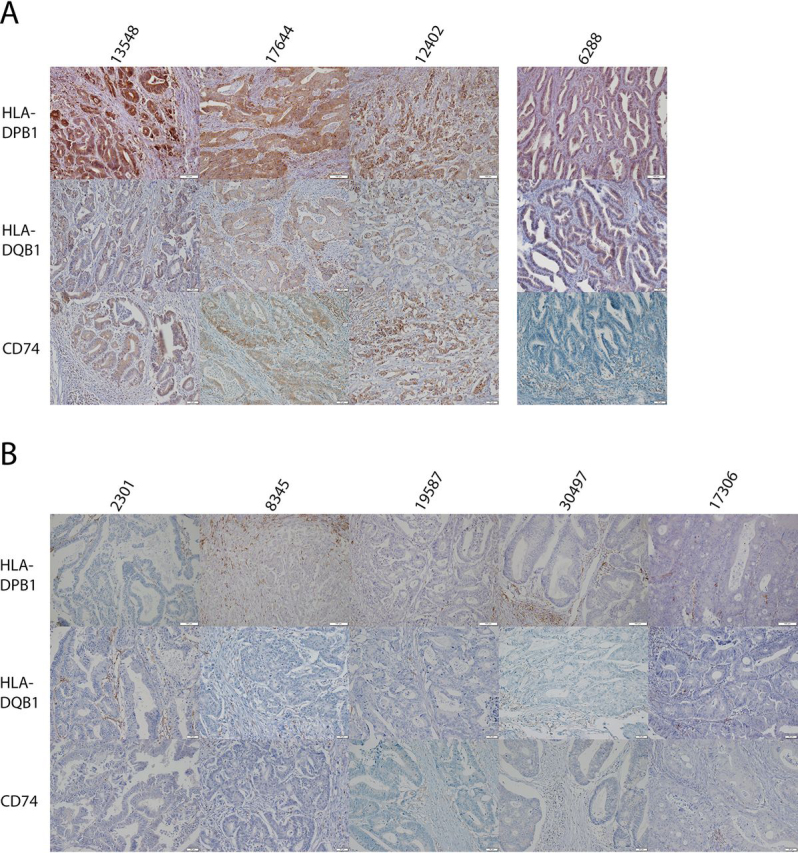
**Immunohistochemistry of MHC type II components shows expression by MSI-H tumor cells.***A*, 3 cases of MSI-H tumor predicted to be with high and low (6288, separated) MHC-II related protein expression. Immunohistochemistry shows that the three high cases are positive for all three proteins (*brown* is positive), whereas case 6288 is weakly-moderately positive for HLA-DPB1\DQB1, but negative to CD74. Positive immune cells can be seen in between the tumor nests. *B*, The tumor nests of five microsatellite stable cases are negative; however, positive immune cells are seen in the stroma.

To differentiate between the possibilities, and to validate our results, we analyzed the spatial distribution of several protein candidates by immunohistochemistry (IHC). MHC-II proteins were chosen as they are not commonly expressed by epithelial cells, and because MSI-H tumors cells have been reported to be positive (39). We analyzed five MSS and four MSI-H tumors, including one MSI-H tumor that our proteomic analysis predicted it to be with medium MHC-II expression (sample 6288; Fig. 4*A*).

Our IHC assay confirmed that the stromal lymphocytes exhibit HLA-DPB1, HLA-DQB1 and CD74 expression in both MSS and MSI-H tumors, acting as an internal positive control. However, tumor cells with diffuse, cytoplasmatic and membranous positivity were found only in MSI-H samples (Fig. 4).

The IHC results indicate that the immunological signature is driven mainly by the tumor cells. Furthermore, it highlights a robust expression of MHC-II, the ligand of the immune checkpoint LAG-3, by the MSI-H tumor cells (40, 41).

##### Only STAT1 Levels Are Linked to Interferon-γ Signaling

The human STAT protein family has 7 members (42). Upon Interferon-γ exposure phospho-STAT1 and STAT3 are increased in amount, translocate to the cell nucleus where they act as transcription activators (43, 44). However, information about the connection of total STAT to biological processes is mostly unknown (13, 44). To identify the proteins that follow the different STAT proteins expression pattern we computed a Pearson's correlation matrix at the individual protein level (1664 proteins).

**Fig. 5:**
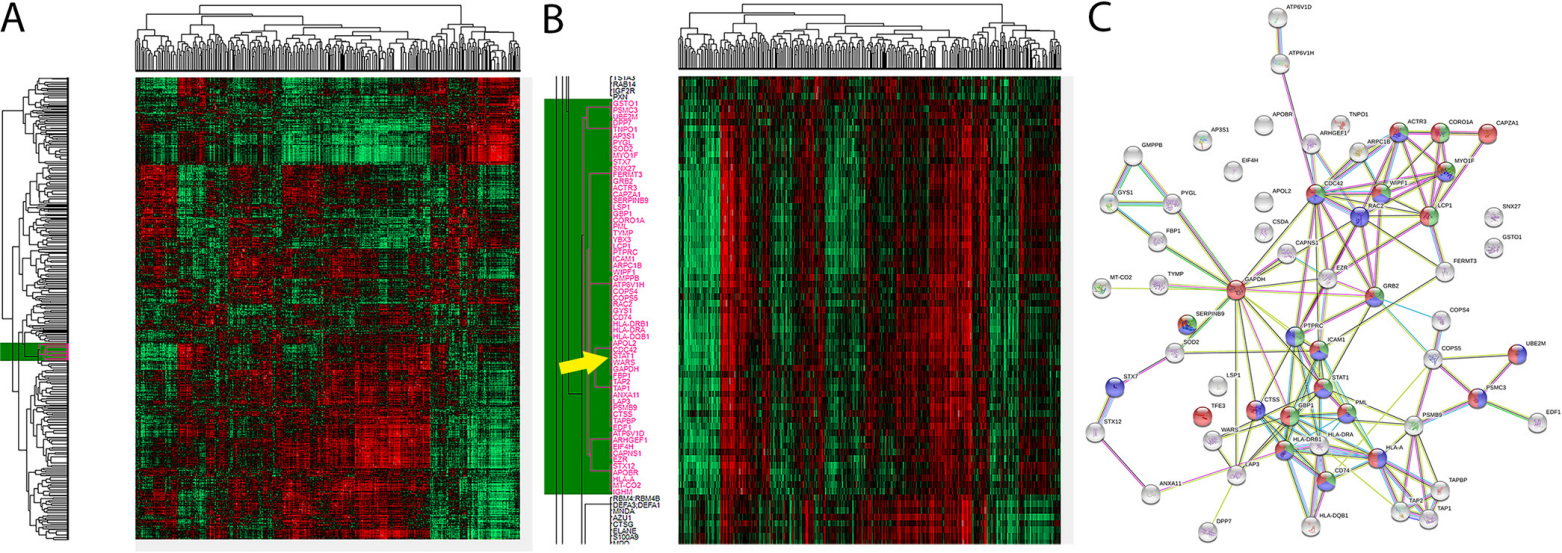
**Unsupervised hierarchical clustering of Pearson's correlation matrix (1664 proteins).** The Pearson's correlation of each protein combination has plotted as a heat map (Euclidean distance-based clustering). *A*, The entire matrix showing all different clusters. The STAT1 cluster is marked in green. *B*, STAT1's 51 protein cluster in magnification. The differences at the borders of the STAT1 cluster are clearly seen. *C*, Graphical representation of the STRING analysis. The 51 proteins have statistically significant interactions increase with 134 edges (expected number = 43). Functional enrichment shows that: 21 proteins (marked *red*) are part of immune response (Red filling, GO6955; 7.07e^−08^); 18 proteins (marked *blue*) are connected to regulation of immune response (GO50776; FDR 2.16e^−08^); 15 proteins (marked *green*) are immune effector process (FDR 9.23e^−09^).

Using unsupervised hierarchical clustering we have found that 61 proteins clustered close to STAT1 (Fig. 5; and supplemental Table S3). STRING analysis found it highly enriched (enrichment *p*-Value < 10^−16^). The top three GO functional enriched processes were immune related (21/61 proteins; Fig. 5*C*). Furthermore, total STAT1 levels are linked to the Interferon-γ-mediated signaling pathway (GO60333; 6 proteins, FDR 4.65e^−05^) and the phagosome (KEGG4145; 11 proteins, FDR 1.1e^−10^). We also identified proteins that connect to MHC type-I (HLA-A, TAP1, TAP2, and TAPBP), and type-II (CD74, HLA-DQB1, HLA-DRB1, CTSS).

STAT3 was reported to mediate Interferon-γ activation and immune checkpoint expression (45, 46). Using the same correlation matrix, we found 54 proteins that clustered around STAT3. STRING analysis found interaction enrichment between them (enrichment *p*-Value 2.5e^−05^) (supplemental Fig. S2). However, the biological processes related to STAT3 appear to be different from STAT1's, and no correlation to immunological pathways were noted (supplemental Table S3).

To expand our understanding of STAT proteins related signatures, we created a correlation matrix with 2345 proteins (quantified ≥70% of samples). Using this correlation matrix, we were able to investigate also STAT-5 and -6 protein clusters. Although STRING analysis found statistically significant interactions within the STAT5 and STAT6 clusters (*p*-Value <1e^−16^ and 1.1e^−14^, respectively), they were linked to biological processes, which were very different of STAT1's immune related processes (supplemental Fig. S3; and supplemental Table S4).

We conclude that the correlation matrix of protein levels contain abundant data about important biological processes in CRC. We showed that each of the four STAT protein expression is associated with different proteins and biological processes. Significantly, our data suggest that *in vivo* only STAT1 protein levels appear to correlate with the same immunological processes that differentiate MSI-H from MSS tumors.

##### Long, but Not Short Exposure to Interferon-γ Differentiates STAT1 from STAT3

Although Interferon-γ is a key factor in MSI-H tumors (39, 47, 48), the changes Interferon-γ induces in CRC in the proteomic level are still poorly understood.

First, we cultured DLD1 and RKO, both micro-satellite instable, human CRC cell lines, in the presence of increasing amounts of Interferon-γ (supplemental Fig. S4). Interestingly, we observed an increase in the phospho- and total- amounts of STAT1 and STAT3. We decided to continue with 10 ng/ml (50 units/ml).

**Fig. 6:**
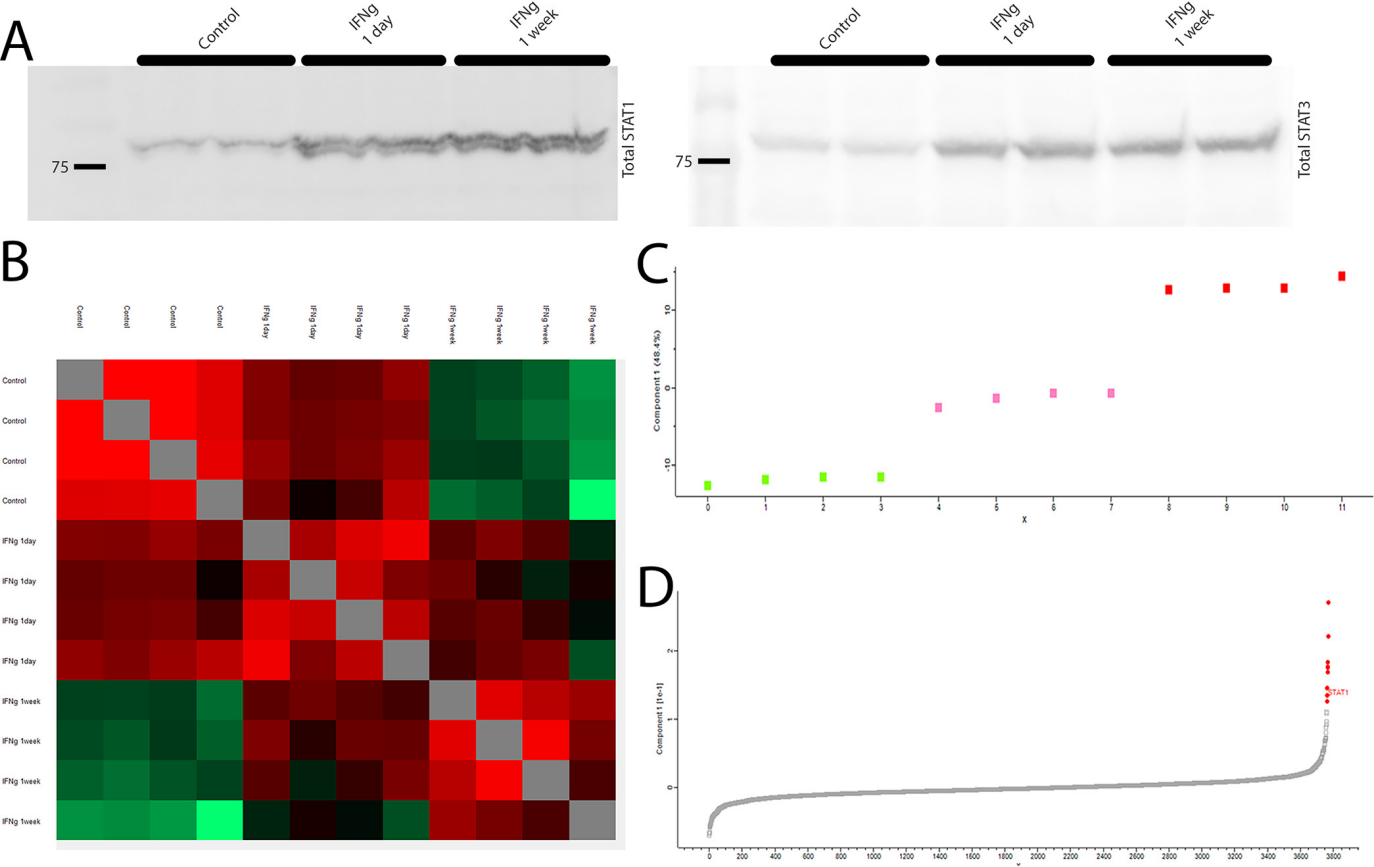
**STAT1, but not STAT3, levels continue to rise because of long IFNg exposure.***A*, Western blot analysis of DLD1 cell line; STAT-1 and STAT-3 levels. Total STAT-1 increase with time, whereas total STAT3 seem to plateau after a day of exposure. *B*, the DLD1 proteome Pearson's correlation matrix. *C*, PCA analysis (comp.1) clearly separates the three experimental arms. (Green, Pink and *red* rectangles, as control, 1-day-, and a week- of interferon exposure, respectively). *D*, The proteins that contribute the most for the PCA scoring (marked in *red*). STAT1 is marked.

We then investigated the STAT1 and 3 protein levels after short- or long-Interferon-γ exposure, a day and a week, respectively. Significantly, total STAT3 levels plateaued after a day, whereas STAT1 levels continued to rise between 1 day and 1 week exposures (Fig. 6*A*; and supplemental Fig. S4B), which suggested a different temporal regulation.

##### In-Vitro Only STAT1 Levels Connect to Long Interferon-γ Exposure

MSI-H tumors evolve for long periods under immunological pressure and cytokine exposure. MSI-H tumors display higher CD8^+^ T cell infiltration, and a vigorous immune microenvironment (11, 49). To evade the hostile microenvironment, MSI-H tumors express a wide plethora of immune-checkpoints, as PD-1, PD-L1, lymphocyte activating 3 (LAG3) and indoleamine 2,3-dioxygenase 1 (IDO1) (11, 50).

Because of the data mentioned above, we investigated the temporal proteomic changes Interferon-γ induces in cancer cells, focusing particularly on the long-term changes. To gain a systematic view on the subject, we analyzed the proteomes of DLD1 exposed to Interferon-γ for a day, a week, and of control cells. Using label-free shotgun proteomics we quantified 3771 proteins in all samples (with at least 3 peptides), among them STAT1 and 3.

We computed the Pearson's correlation between the samples. We noted that the control and the IFN-g 1-week groups showed the biggest difference, with the INF-g 1 day group in between (Fig. 6*B*). This suggested that the proteomes of the three groups are different, and that there are significant temporal related changes. Indeed, unsupervised primary component analysis (PCA comp. 1) matched the experimental design and identified 3 groups (Fig. 6*C*). We found that 9 proteins contributed the most to the PCA (score of more than 1; supplemental Table S5). Significantly, STAT1, TAPBP, TAP-2, HLA-A and HLA-C are among them. STRING analysis found significant interaction enrichment between them (enrichment *p*-Value 1.1e^−11^). Moreover, antigen processing and presentation processes were highly enriched (supplemental Table S5), resembling the biological processes found to be enriched in the MSI-H *versus* MSS tumors.

Finally, we again used a Person's correlation matrix to investigate which proteins follow the STAT proteins' behavior. We found that the four STAT proteins clustered to one major cluster (containing 277 proteins out of 3771 tested), providing some indication that there is a connection between the STAT proteins. However, STRING reported no significant interaction in the proteins clustered around STAT2, 3 and 6 (supplemental Table S6), suggesting that they are not well connected to the changes Interferon-γ induces over time. In sharp contrast, STAT1 showed a highly enriched network (supplemental Table S5) (PPI enrichment *p*-value <10^−16^). Importantly, the proteins correlated to STAT1 were highly enriched for biological processes such as Interferon and antigen processing and presentation (supplemental Table S6).

##### Interferon-γ Exposure Triggers Complex Temporal Proteome Changes

Our previous findings suggested that proteins levels changed differently over time. Thus, we investigated all of the proteins showing a statistically significant change between any two groups (multiple-sample test, ANOVA FDR = 0.01, S = 0.1). Strikingly, we found that 16.1% of the proteome (608/3771 proteins) was expressed differentially.

**Fig. 7:**
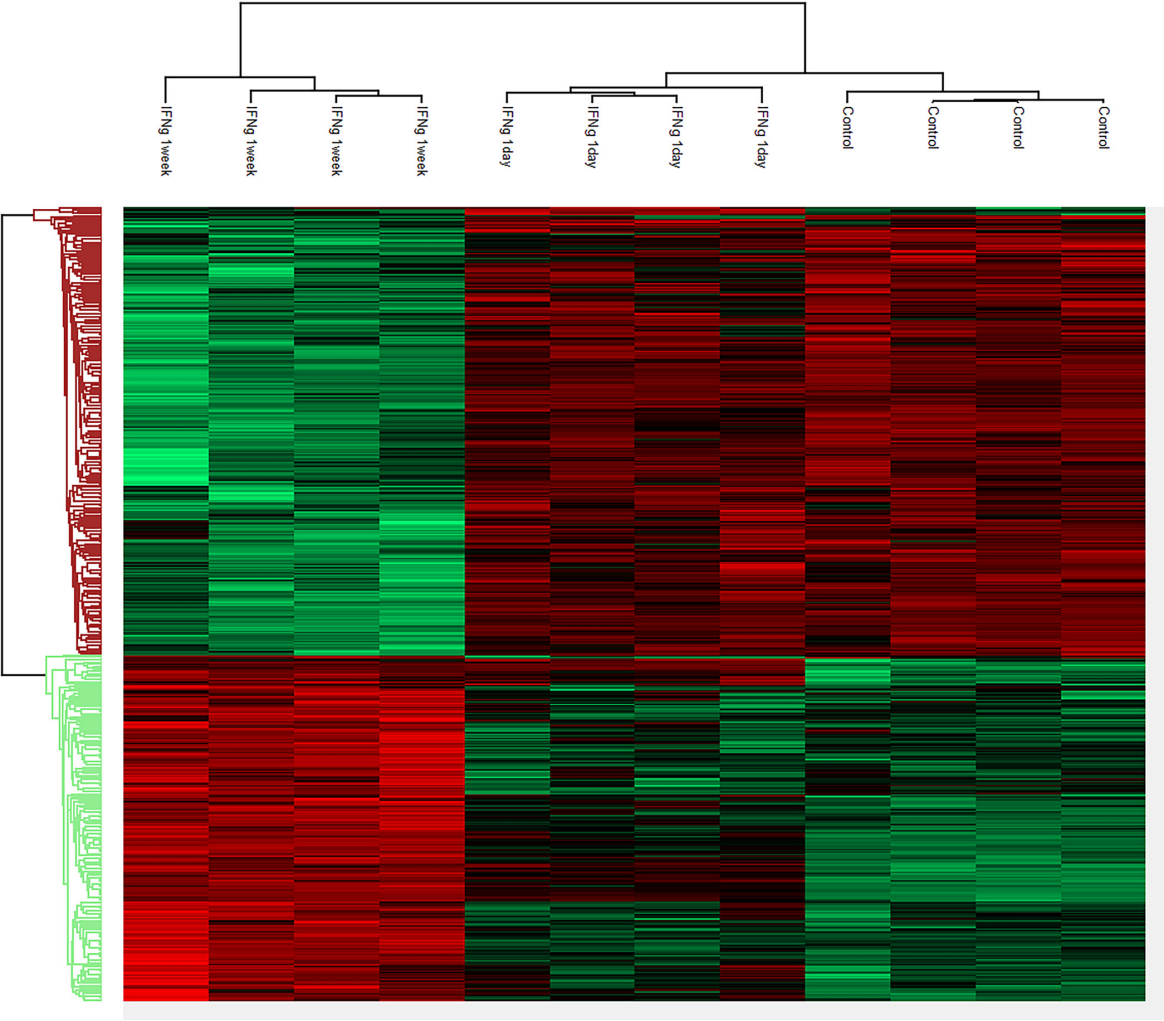
**Supervised hierarchial clustering of all differentially expressed proteins (608 proteins).** The supervised hierarchical clustering clearly highlights that a significant change in the proteome occurs after a week of exposure to IFNg, reflected by the segregation of the 1 week IFNg exposure from the other two groups. However, complex temporal behavior of the proteome is clear, especially in the proteins up-regulated by interferon (*green* cluster).

Hierarchical clustering of these proteins clearly displayed the temporal changes and highlighted a complex temporal behavior. Two main clusters of proteins were identified, 343 and 265 proteins in each cluster (Fig. 7*A*; supplemental Table S7). However, within these clusters a complex pattern of expression can be seen.

## DISCUSSION

Today's proteomics studies have the capacity to capture and analyze the proteome in great depth and accuracy (51). Indeed, significant improvements in the MS machines (52, 53) and label-free quantification bioinformatics have been made (54). However, the usage of formalin-fixed paraffin embedded tissues, the most common clinical material in the pathology departments, is still complicated. One of the main problems is the wide variability of formalin fixation times observed in the clinics (5).

For this reason, we optimized a novel proteome extraction method suitable for wide fixation times. Furthermore, sample preparation steps were simplified to increase performance, and the elevated temperature and pressure seem to solubilize proteins very efficiently. Our TOP method uses only nonhazardous, environmentally friendly reagents, as opposed to the traditional method, which uses organic solvents. As a result, proteome extraction is done in about 3 h after starting from FFPE cuts, without the need for a fume hood.

Most researchers divide CRC into two main groups, MSS and MSI-H tumors (30). Indeed, recently the FDA-approved immunooncology treatments for MSI-H tumors across multiple indications in the first ever tumor type agnostic manner (55), suggesting a significant role for the immune system in the MSI-H cancers.

Here we demonstrate that optimized clinical proteomics pipeline might help to generate novel fundamental biological insights. Using the TOP method and only nineteen FFPE archival tissues, we were able to identify and quantify over 4100 proteins, of which 1664 were identified in all samples. Unsupervised hierarchical clustering of our data followed the MSS and MSI-H groups, pointing that the proteomes of MSS and MSI-H groups are different.

Further investigation showed that most of the proteins overexpressed by MSI-H tumors are connected to numerous immune system related processes, among them antigen processing and presentation. Importantly, we noted the overexpressed proteins in most of MSI-H patients, suggesting a presence of a preserved biological program. Using immunohistochemistry, we were able to show that the tumor cells, and not the stroma, are responsible for the aberrantly expression of MHC-II, a ligand of the immune-check point protein LAG-3.

Significantly, we noted that STAT1 is overexpressed by the MSI-H tumor cells. Further insight into STAT1's role came from the protein correlation matrix. We were able to show that *in vivo* only STAT1 levels, but not levels of other STAT proteins, are correlated to these immunological processes.

These observations were confusing because STAT1 is considered a tumor suppressor in CRC (43). This view stems from few observations. First, STAT1 was linked to anti-proliferative and pro-apoptotic signaling pathways (56). Specifically, in CRC, nuclear STAT1 was shown to be a good prognostic marker (14), whereas high levels of cytoplasmic STAT1 correlated with shorter survival in early stage CRC, particularly of the microsatellite instability cases (57). In sharp contrast, STAT1 was shown to be an immune tumor promoter for leukemia development. Importantly, STAT1 null tumor cells showed enhanced natural killer cell lysis because of their low protein expression of antigen presentation molecules. Furthermore, upon leukemia progression the STAT1 null cells acquired an increased amount of MHC-I (58).

To better understand the roles of Interferon-γ and STAT proteins in CRC, we turned to an *in vitro* system. Our hypothesis was that MSI-H tumor cells evolve under immunological pressure for long periods. Furthermore, we know that Interferon-γ is important for this system, and that all nucleated cells express Interferon-receptors (59). Thus, we decided to investigate the poorly understood temporal proteomics changes induced by Interferon-γ.

PCA analysis of the DLD1 proteomes showed that the proteome changes reflect the length of exposure to Interferon-γ. Significantly, by PCA analysis we identified STAT1 and several other proteins that correlate with these changes. Further substantiating evidence came from the protein correlation matrix of the *in vitro* experiment. Like what was seen in the patients: antigen presentation, the proteasome, and various immunological processes were highly enriched. In sharp contrast, the proteins that clustered around the other STAT proteins showed no evidence of interactions, nor linkage to immune-related processes. The fact that these *in vitro* results emulate the *in vivo* results, substantiates the linkage of STAT1 levels with the time-dependent Interferon-γ proteomic changes and the immunological program seen in MSI-H CRC tumors.

Our data clearly points that in CRC, the role of STAT1 is complex and perhaps pathogenesis dependent. The MSI-H neoplastic cells abnormally express MHC-II complex, and STAT1 modulates CIITA transcription, a key inducer of MHC-II expression by Interferon-γ (60, 61). MHC-II complex is a known ligand of the immune checkpoint, lymphocyte activation gene-3 (LAG-3, CD223). Furthermore, we observed a high correlation between the mRNAs of STAT1 to LAG-3 and PD-L1 (TCGA CRC data, data not shown). Taken together, our data suggests a link between high STAT1 levels and immune evasion, which supports in turn that STAT1 is an immune-tumor promoter in MSI-H CRC, but not in MSS CRC. However, further studies are needed to substantiate this point.

In summary, clinical proteomics has matured significantly, and optimized pipelines will continue to improve our understanding of human diseases. Using these tools we show that MSI-H CRC tumors express high levels of STAT1. Our findings points toward a preserved, constant primary immune escape mechanism of CRC MSI-H tumors. We hope that our findings will enable further drug development.

## DATA AVAILABILITY

The MS proteomics data have been deposited to the ProteomeXchange Consortium via the PRIDE partner repository with the data set identifiers PXD019252, PXD019254, and PXD017821.

10.13039/501100003977Israel Science Foundation (ISF) (2131/15) to Gilad W. Vainer
10.13039/501100003975Israel Cancer Association (ICA) (20181134) to Gilad W. Vainer
10.13039/501100001736German-Israeli Foundation for Scientific Research and Development (GIF) (I-1444-201.2/2017) to Elez D. Vainer, and Gilad W. Vainer
10.13039/501100001736German-Israeli Foundation for Scientific Research and Development (GIF) (I-1444-201.2/2017) to Elez D. Vainer, and Gilad W. Vainer
10.13039/501100000833Rosetrees Trust (1) to Gilad W. Vainer

## References

[1] Wiśniewski J.R., Duś-Szachniewicz K., Ostasiewicz P., Ziółkowski P., Rakus D., Mann M. (2015). Absolute proteome analysis of colorectal mucosa, adenoma, and cancer reveals drastic changes in fatty acid metabolism and plasma membrane transporters. J. Proteome Res.

[2] Ostasiewicz P., Zielinska D.F., Mann M., Wiśniewski J.R. (2010). Proteome, phosphoproteome, and N-glycoproteome are quantitatively preserved in formalin-fixed paraffin-embedded tissue and analyzable by high-resolution mass spectrometry. J. Proteome Res.

[3] Giusti L., Angeloni C., Lucacchini A. (2019). Update on proteomic studies of formalin-fixed paraffin-embedded tissues. Expert Rev. Proteomics.

[4] Hammond M.E., Hayes D.F., Dowsett M. (2010). American Society of Clinical Oncology/College of American Pathologists guideline recommendations for immunohistochemical testing of estrogen and progesterone receptors in breast cancer. Arch Pathol Lab Med.

[5] Wolff C., Schott C., Porschewski P., Reischauer B., Becker K.-F. (2011). Successful protein extraction from over-fixed and long-term stored formalin-fixed tissues. PLoS ONE.

[6] Ferlay J., Soerjomataram I., Dikshit R., Eser S., Mathers C., Rebelo M., Parkin D.M., Forman D., Bray F. (2015). Cancer incidence and mortality worldwide: sources, methods and major patterns in GLOBOCAN 2012. Int. J. Cancer.

[7] Cancer Genome Atlas N (2012). Comprehensive molecular characterization of human colon and rectal cancer. Nature.

[8] Ryan E., Sheahan K., Creavin B., Mohan H.M., Winter D.C. (2017). The current value of determining the mismatch repair status of colorectal cancer: A rationale for routine testing. Crit. Rev. Oncol. Hematol.

[9] Alexandrov L.B., Nik-Zainal S., Wedge D.C., Aparicio S.A.J.R., Behjati S., Biankin A.V., Bignell G.R., Bolli N., Borg A., Børresen-Dale A.-L., Boyault S., Burkhardt B., Butler A.P., Caldas C., Davies H.R., Desmedt C., Eils R., Eyfjörd J.E., Foekens J.A., Greaves M., Hosoda F., Hutter B., Ilicic T., Imbeaud S., Imielinski M., Imielinsk M., Jäger N., Jones D.T.W., Jones D., Knappskog S., Kool M., Lakhani S.R., López-Otín C., Martin S., Munshi N.C., Nakamura H., Northcott P.A., Pajic M., Papaemmanuil E., Paradiso A., Pearson J.V., Puente X.S., Raine K., Ramakrishna M., Richardson A.L., Richter J., Rosenstiel P., Schlesner M., Schumacher T.N., Span P.N., Teague J.W., Totoki Y., Tutt A.N.J., Valdés-Mas R., van Buuren M.M., van 't Veer L., Vincent-Salomon A., Waddell N., Yates L.R., Zucman-Rossi J., Futreal P.A., McDermott U., Lichter P., Meyerson M., Grimmond S.M., Siebert R., Campo E., Shibata T., Pfister S.M., Campbell P.J., Stratton M.R, ICGC PedBrain (2013). Signatures of mutational processes in human cancer. Nature.

[10] Nebot-Bral L., Brandao D., Verlingue L., Rouleau E., Caron O., Despras E., El-Dakdouki Y., Champiat S., Aoufouchi S., Leary A., Marabelle A., Malka D., Chaput N., Kannouche P.L. (2017). Hypermutated tumours in the era of immunotherapy: The paradigm of personalised medicine. Eur. J. Cancer.

[11] Llosa N.J., Cruise M., Tam A., Wicks E.C., Hechenbleikner E.M., Taube J.M., Blosser R.L., Fan H., Wang H., Luber B.S., Zhang M., Papadopoulos N., Kinzler K.W., Vogelstein B., Sears C.L., Anders R.A., Pardoll D.M., Housseau F. (2015). The vigorous immune microenvironment of microsatellite instable colon cancer is balanced by multiple counter-inhibitory checkpoints. Cancer Discov.

[12] Vogelstein B., Papadopoulos N., Velculescu V.E., Zhou S., Diaz L.A., Kinzler K.W. (2013). Cancer genome landscapes. Science.

[13] Friedrich K., Dolznig H., Han X., Moriggl R. (2017). Steering of carcinoma progression by the YIN/YANG interaction of STAT1/STAT3. Biosci. Trends.

[14] Simpson J.A.D., Al-Attar A., Watson N.F.S., Scholefield J.H., Ilyas M., Durrant L.G. (2010). Intratumoral T cell infiltration, MHC class I and STAT1 as biomarkers of good prognosis in colorectal cancer. Gut.

[15] Gordziel C., Bratsch J., Moriggl R., Knösel T., Friedrich K. (2013). Both STAT1 and STAT3 are favourable prognostic determinants in colorectal carcinoma. Br. J. Cancer.

[16] Klupp F., Diers J., Kahlert C., Neumann L., Halama N., Franz C., Schmidt T., Lasitschka F., Warth A., Weitz J., Koch M., Schneider M., Ulrich A. (2015). Expressional STAT3/STAT5 ratio is an independent prognostic marker in colon carcinoma. Ann. Surg. Oncol.

[17] Corvinus F.M., Orth C., Moriggl R., Tsareva S.A., Wagner S., Pfitzner E.B., Baus D., Kaufmann R., Huber L.A., Zatloukal K., Beug H., Ohlschläger P., Schütz A., Halbhuber K.-J., Friedrich K. (2005). Persistent STAT3 activation in colon cancer is associated with enhanced cell proliferation and tumor growth. Neoplasia.

[18] Tsareva S.A., Moriggl R., Corvinus F.M., Wiederanders B., Schütz A., Kovacic B., Friedrich K. (2007). Signal transducer and activator of transcription 3 activation promotes invasive growth of colon carcinomas through matrix metalloproteinase induction. Neoplasia.

[19] Grivennikov S., Karin E., Terzic J., Mucida D., Yu G.-Y., Vallabhapurapu S., Scheller J., Rose-John S., Cheroutre H., Eckmann L., Karin M. (2009). IL-6 and Stat3 are required for survival of intestinal epithelial cells and development of colitis-associated cancer. Cancer Cell.

[20] Quante M., Varga J., Wang T.C., Greten F.R. (2013). The gastrointestinal tumor microenvironment. Gastroenterology.

[21] Musteanu M., Blaas L., Mair M., Schlederer M., Bilban M., Tauber S., Esterbauer H., Mueller M., Casanova E., Kenner L., Poli V., Eferl R. (2010). Stat3 is a negative regulator of intestinal tumor progression in Apc(Min) mice. Gastroenterology.

[22] Wisniewski J.R. (2013). Proteomic sample preparation from formalin fixed and paraffin embedded tissue. J Vis Exp.

[23] Wiśniewski J.R., Ostasiewicz P., Mann M. (2011). High recovery FASP applied to the proteomic analysis of microdissected formalin fixed paraffin embedded cancer tissues retrieves known colon cancer markers. J. Proteome Res.

[24] Wiśniewski J.R., Zougman A., Nagaraj N., Mann M. (2009). Universal sample preparation method for proteome analysis. Nat. Methods.

[25] Cox J., Mann M. (2008). MaxQuant enables high peptide identification rates, individualized p.p.b.-range mass accuracies and proteome-wide protein quantification. Nat. Biotechnol.

[26] Cox J., Neuhauser N., Michalski A., Scheltema R.A., Olsen J.V., Mann M. (2011). Andromeda: a peptide search engine integrated into the MaxQuant environment. J. Proteome Res.

[27] Shi S.-R., Taylor C.R., Fowler C.B., Mason J.T. (2013). Complete solubilization of formalin-fixed, paraffin-embedded tissue may improve proteomic studies. Proteomics. Clin. Appl.

[28] Gurjao C., Liu D., Hofree M., AlDubayan S.H., Wakiro I., Su M.-J., Felt K., Gjini E., Brais L.K., Rotem A., Rosenthal M.H., Rozenblatt-Rosen O., Rodig S., Ng K., Van Allen E.M., Corsello S.M., Ogino S., Regev A., Nowak J.A., Giannakis M. (2019). Intrinsic resistance to immune checkpoint blockade in a mismatch repair-deficient colorectal cancer. Cancer Immunol. Res.

[29] Das S., Berlin J., Cardin D. (2018). Harnessing the immune system in pancreatic cancer. Curr Treat Options Oncol.

[30] Zhang B., Wang J., Wang X., Zhu J., Liu Q., Shi Z., Chambers M.C., Zimmerman L.J., Shaddox K.F., Kim S., Davies S.R., Wang S., Wang P., Kinsinger C.R., Rivers R.C., Rodriguez H., Townsend R.R., Ellis M.J.C., Carr S.A., Tabb D.L., Coffey R.J., Slebos R.J.C., Liebler D.C, NCI CPTAC (2014). Proteogenomic characterization of human colon and rectal cancer. Nature.

[31] Edwards N.J., Oberti M., Thangudu R.R., Cai S., McGarvey P.B., Jacob S., Madhavan S., Ketchum K.A. (2015). The CPTAC data portal: a resource for cancer proteomics research. J. Proteome Res.

[32] Szklarczyk D., Gable A.L., Lyon D., Junge A., Wyder S., Huerta-Cepas J., Simonovic M., Doncheva N.T., Morris J.H., Bork P., Jensen L.J., Mering C.V. (2019). STRING v11: protein-protein association networks with increased coverage, supporting functional discovery in genome-wide experimental datasets. Nucleic Acids Res.

[33] Sikorski K., Czerwoniec A., Bujnicki J.M., Wesoly J., Bluyssen H.A.R. (2011). STAT1 as a novel therapeutical target in pro-atherogenic signal integration of IFNgamma, TLR4 and IL-6 in vascular disease. Cytokine Growth Factor Rev.

[34] Dupuis S., Döffinger R., Picard C., Fieschi C., Altare F., Jouanguy E., Abel L., Casanova J.L. (2000). Human interferon-gamma-mediated immunity is a genetically controlled continuous trait that determines the outcome of mycobacterial invasion. Immunol. Rev.

[35] Blaszczyk K., Nowicka H., Kostyrko K., Antonczyk A., Wesoly J., Bluyssen H.A.R. (2016). The unique role of STAT2 in constitutive and IFN-induced transcription and antiviral responses. Cytokine Growth Factor Rev.

[36] Fridrichova I. (2006). New aspects in molecular diagnosis of Lynch syndrome (HNPCC). Cancer Biomark.

[37] Toh J.W.T., de Souza P., Lim S.H., Singh P., Chua W., Ng W., Spring K.J. (2016). The potential value of immunotherapy in colorectal cancers: review of the evidence for programmed death-1 inhibitor therapy. Clin Colorectal Cancer.

[38] Solinas C., Pusole G., Demurtas L., Puzzoni M., Mascia R., Morgan G., Giampieri R., Scartozzi M. (2017). Tumor infiltrating lymphocytes in gastrointestinal tumors: Controversies and future clinical implications. Crit. Rev. Oncol. Hematol.

[39] Michel S., Linnebacher M., Alcaniz J., Voss M., Wagner R., Dippold W., Becker C., von Knebel Doeberitz M., Ferrone S., Kloor M. (2010). Lack of HLA class II antigen expression in microsatellite unstable colorectal carcinomas is caused by mutations in HLA class II regulatory genes. Int. J. Cancer.

[40] Baixeras E., Huard B., Miossec C., Jitsukawa S., Martin M., Hercend T., Auffray C., Triebel F., Piatier-Tonneau D. (1992). Characterization of the lymphocyte activation gene 3-encoded protein. A new ligand for human leukocyte antigen class II antigens. J. Exp. Med.

[41] Grosso J.F., Kelleher C.C., Harris T.J., Maris C.H., Hipkiss E.L., De Marzo A., Anders R., Netto G., Getnet D., Bruno T.C., Goldberg M.V., Pardoll D.M., Drake C.G. (2007). LAG-3 regulates CD8+ T cell accumulation and effector function in murine self- and tumor-tolerance systems. J. Clin. Invest.

[42] Ihle J.N. (1996). STATs: signal transducers and activators of transcription. Cell.

[43] Vogel T.P., Milner J.D., Cooper M.A. (2015). The Ying and Yang of STAT3 in Human Disease. J. Clin. Immunol.

[44] Cheon H., Stark G.R. (2009). Unphosphorylated STAT1 prolongs the expression of interferon-induced immune regulatory genes. Proc. Natl. Acad. Sci. U S A.

[45] Tsai M.H., Pai L.M., Lee C.K. (2019). Fine-tuning of type I interferon response by STAT3. Front. Immunol.

[46] Yoyen-Ermis D., Tunali G., Tavukcuoglu E., Horzum U., Ozkazanc D., Sutlu T., Buyukasik Y., Esendagli G. (2019). Myeloid maturation potentiates STAT3-mediated atypical IFN-gamma signaling and upregulation of PD-1 ligands in AML and MDS. Sci. Rep.

[47] Bauer K., Nelius N., Reuschenbach M., Koch M., Weitz J., Steinert G., Kopitz J., Beckhove P., Tariverdian M., von Knebel Doeberitz M., Kloor M. (2013). T cell responses against microsatellite instability-induced frameshift peptides and influence of regulatory T cells in colorectal cancer. Cancer Immunol. Immunother.

[48] Dierssen J.W.F., van Puijenbroek M., Dezentjé D.A., Fleuren G.J., Cornelisse C.J., van Wezel T., Offringa R., Morreau H. (2008). Frequent mutations in the 3'-untranslated region of IFNGR1 lack functional impairment in microsatellite-unstable colorectal tumours. Eur. J. Hum. Genet.

[49] Drescher K.M., Sharma P., Watson P., Gatalica Z., Thibodeau S.N., Lynch H.T. (2009). Lymphocyte recruitment into the tumor site is altered in patients with MSI-H colon cancer. Fam. Cancer.

[50] Havel J.J., Chowell D., Chan T.A. (2019). The evolving landscape of biomarkers for checkpoint inhibitor immunotherapy. Nat. Rev. Cancer.

[51] Mann M., Kulak N.A., Nagaraj N., Cox J. (2013). The coming age of complete, accurate, and ubiquitous proteomes. Mol. Cell.

[52] Kelstrup C.D., Jersie-Christensen R.R., Batth T.S., Arrey T.N., Kuehn A., Kellmann M., Olsen J.V. (2014). Rapid and deep proteomes by faster sequencing on a benchtop quadrupole ultra-high-field Orbitrap mass spectrometer. J. Proteome Res.

[53] Scheltema R.A., Hauschild J.-P., Lange O., Hornburg D., Denisov E., Damoc E., Kuehn A., Makarov A., Mann M. (2014). The Q Exactive HF, a Benchtop mass spectrometer with a pre-filter, high-performance quadrupole and an ultra-high-field Orbitrap analyzer. Mol. Cell. Proteomics.

[54] Cox J., Hein M.Y., Luber C.A., Paron I., Nagaraj N., Mann M. (2014). Accurate proteome-wide label-free quantification by delayed normalization and maximal peptide ratio extraction, termed MaxLFQ. Mol. Cell. Proteomics.

[55] Le D.T., Durham J.N., Smith K.N., Wang H., Bartlett B.R., Aulakh L.K., Lu S., Kemberling H., Wilt C., Luber B.S., Wong F., Azad N.S., Rucki A.A., Laheru D., Donehower R., Zaheer A., Fisher G.A., Crocenzi T.S., Lee J.J., Greten T.F., Duffy A.G., Ciombor K.K., Eyring A.D., Lam B.H., Joe A., Kang S.P., Holdhoff M., Danilova L., Cope L., Meyer C., Zhou S., Goldberg R.M., Armstrong D.K., Bever K.M., Fader A.N., Taube J., Housseau F., Spetzler D., Xiao N., Pardoll D.M., Papadopoulos N., Kinzler K.W., Eshleman J.R., Vogelstein B., Anders R.A., Diaz L.A. (2017). Mismatch repair deficiency predicts response of solid tumors to PD-1 blockade. Science.

[56] Kim H.S., Lee M.S. (2007). STAT1 as a key modulator of cell death. Cell. Signal.

[57] Tanaka A., Zhou Y., Ogawa M., Shia J., Klimstra D.S., Wang J.Y., Roehrl M.H. (2020). STAT1 as a potential prognosis marker for poor outcomes of early stage colorectal cancer with microsatellite instability. PLoS ONE.

[58] Kovacic B., Stoiber D., Moriggl R., Weisz E., Ott R.G., Kreibich R., Levy D.E., Beug H., Freissmuth M., Sexl V. (2006). STAT1 acts as a tumor promoter for leukemia development. Cancer Cell.

[59] Schreiber G. (2017). The molecular basis for differential type I interferon signaling. J. Biol. Chem.

[60] Piskurich J.F., Linhoff M.W., Wang Y., Ting J.P. (1999). Two distinct gamma interferon-inducible promoters of the major histocompatibility complex class II transactivator gene are differentially regulated by STAT1, interferon regulatory factor 1, and transforming growth factor beta. Mol. Cell Biol.

[61] Muhlethaler-Mottet A., Di Berardino W., Otten L.A., Mach B. (1998). Activation of the MHC class II transactivator CIITA by interferon-gamma requires cooperative interaction between Stat1 and USF-1. Immunity.

