# Supplementary material for: Novel Proteome Extraction Method Illustrates a Conserved Immunological Signature of MSI-H Colorectal Tumors
Source: Mol Cell Proteomics. 2020 Nov 25;19(10):1619–31. doi: 10.1074/mcp.RA120.002152 (PMC8015011; doi:10.1074/mcp.RA120.002152)

**Supplementary figure 1. Scatter** **plots and Pearson's correlation scores**. The proteome of each and every sample was plotted against each other. Pearson correlation was calculated for each pair. A very high correlation can be seen across samples. The comparison of the two biological replicates shows very high correlation reflecting the process optimization (scores 0.982 and 0.978, marked by red squares).

**Supplementary figure 2. STAT3 cluster in unsupervised hierarchial clustering of Pearson’s correlation matrix (1664 proteins).** Graphical representation of the STRING analysis.

**Supplementary figure 3. STAT proteins cluster in unsupervised hierarchial clustering of Pearson’s correlation matrix (1664 proteins).** Graphical representation of the STRING analysis. Note the ubiquity of each cluster.

**Supplementary figure 4. DLD1 and RKO western blot analysis.** A. The two MSI-H CRC cell lines have been exposed to increasing amounts of Interferon (1 day). B. The two MSI-H CRC cell lines have been exposed to Interferon for different time periods. Note that both cell lines show a plateau of STAT3 levels after a day, while STAT1 continue to increase its amounts.

Sup fig.1


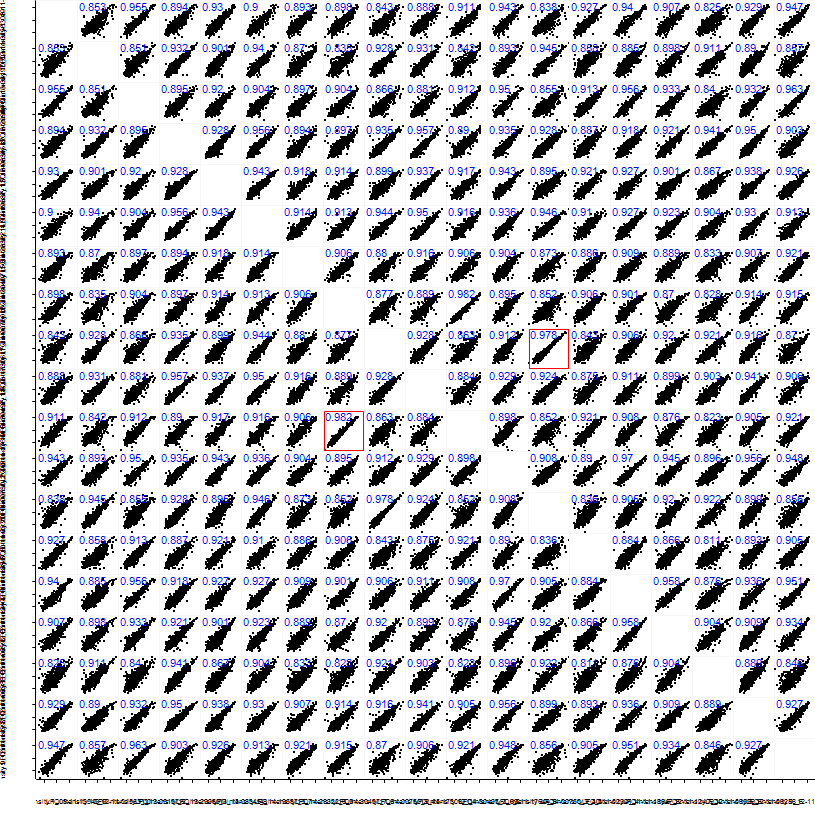


Sup fig.2


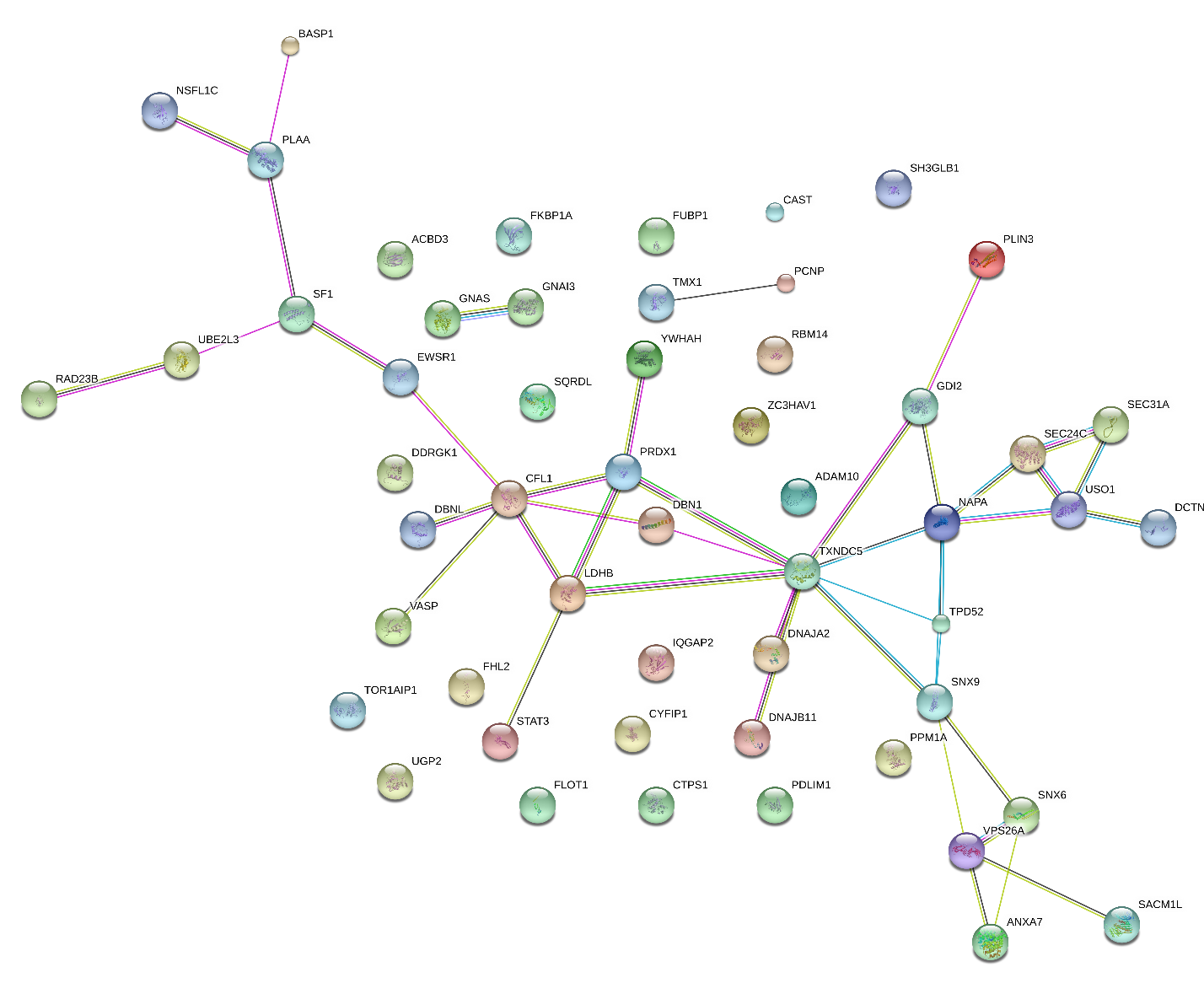


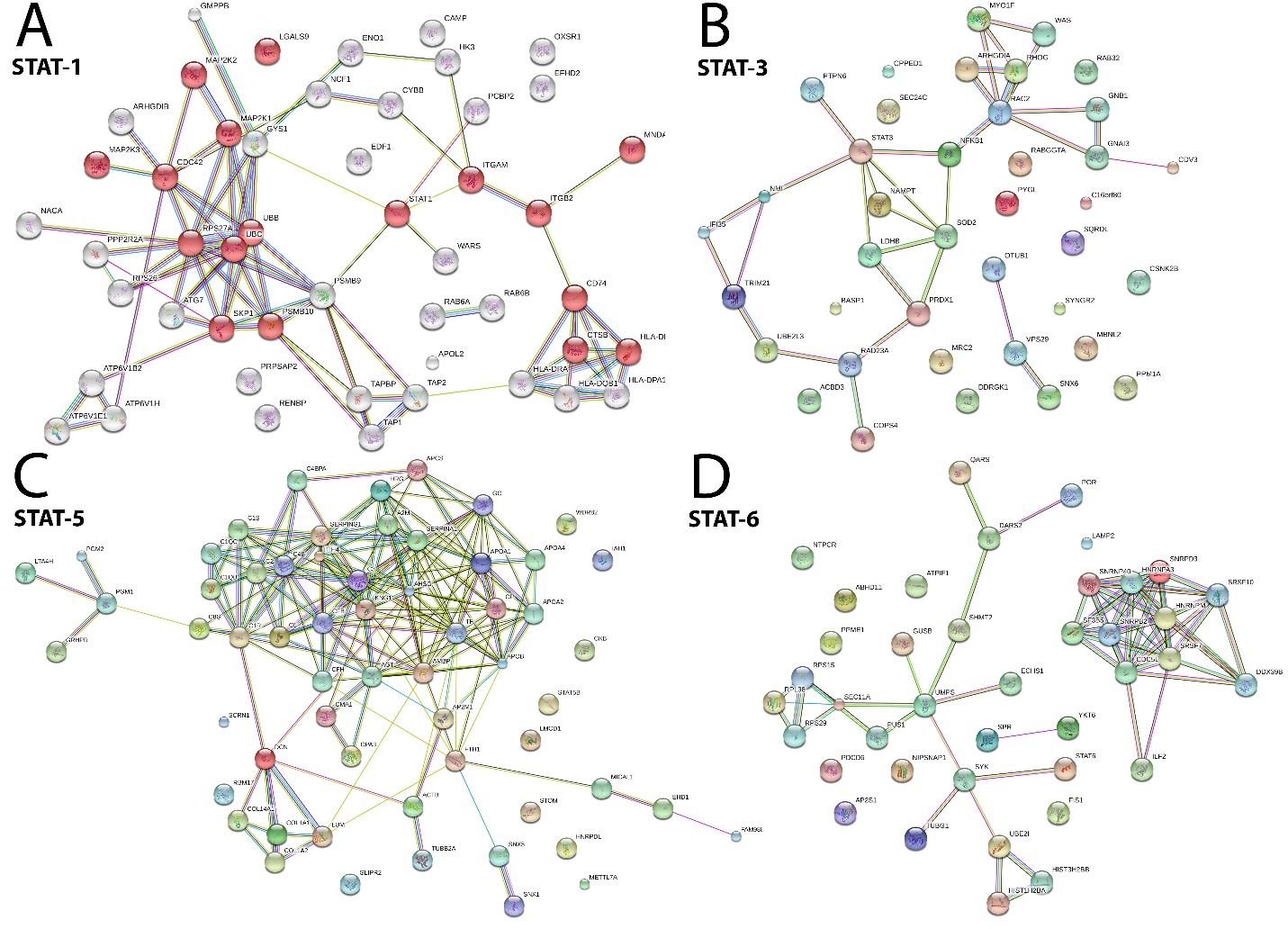


Sup fig.3

Sup fig.4


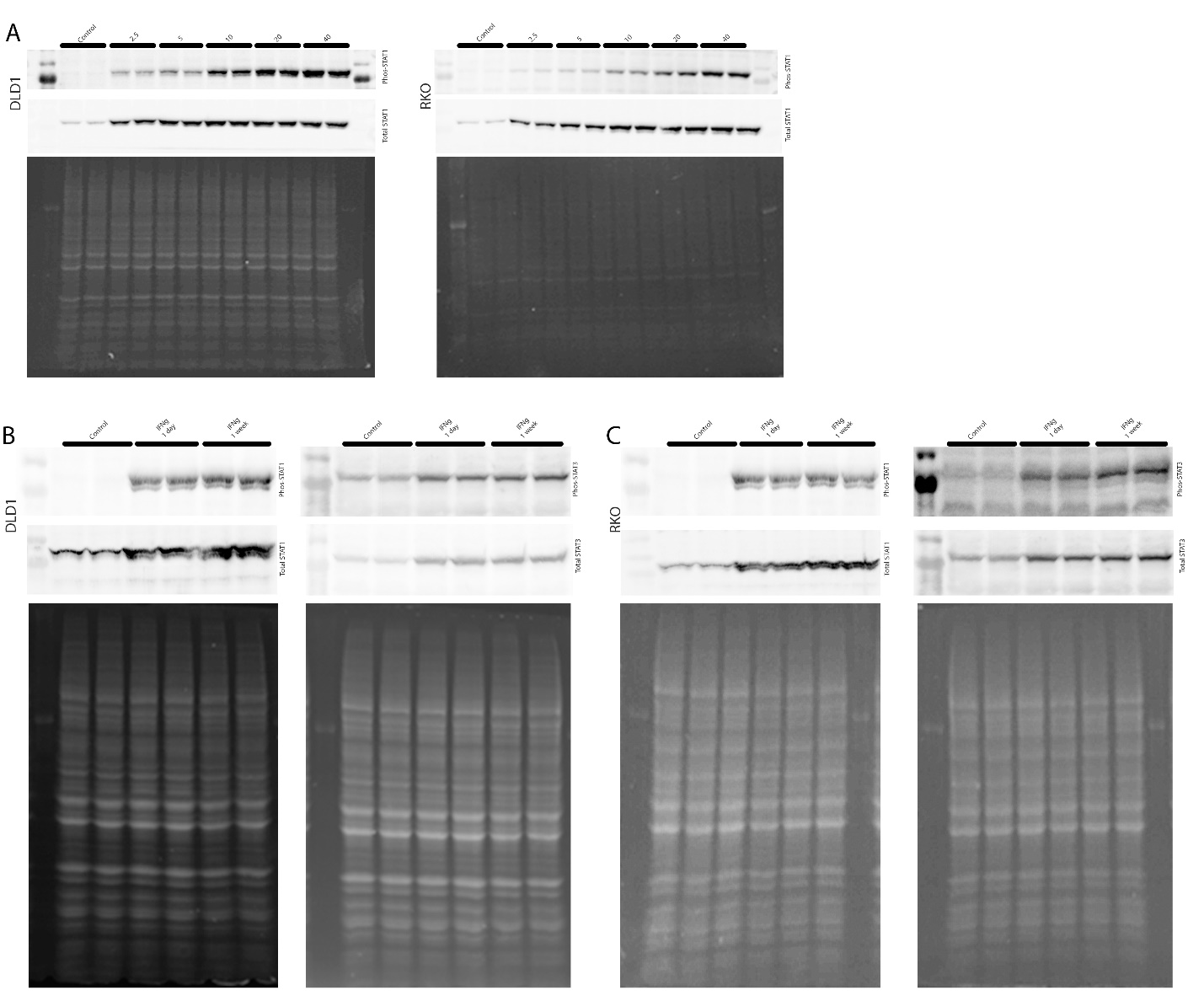

Supplement: Supplementary file 1 [file mmc1.zip › 161224_1_supp_556354_qcjrjv.docx]
