# Supplementary figures and images for: Novel Proteome Extraction Method Illustrates a Conserved Immunological Signature of MSI-H Colorectal Tumors
Source: Mol Cell Proteomics. 2020 Nov 25;19(10):1619–31. doi: 10.1074/mcp.RA120.002152 (PMC8015011; doi:10.1074/mcp.RA120.002152)

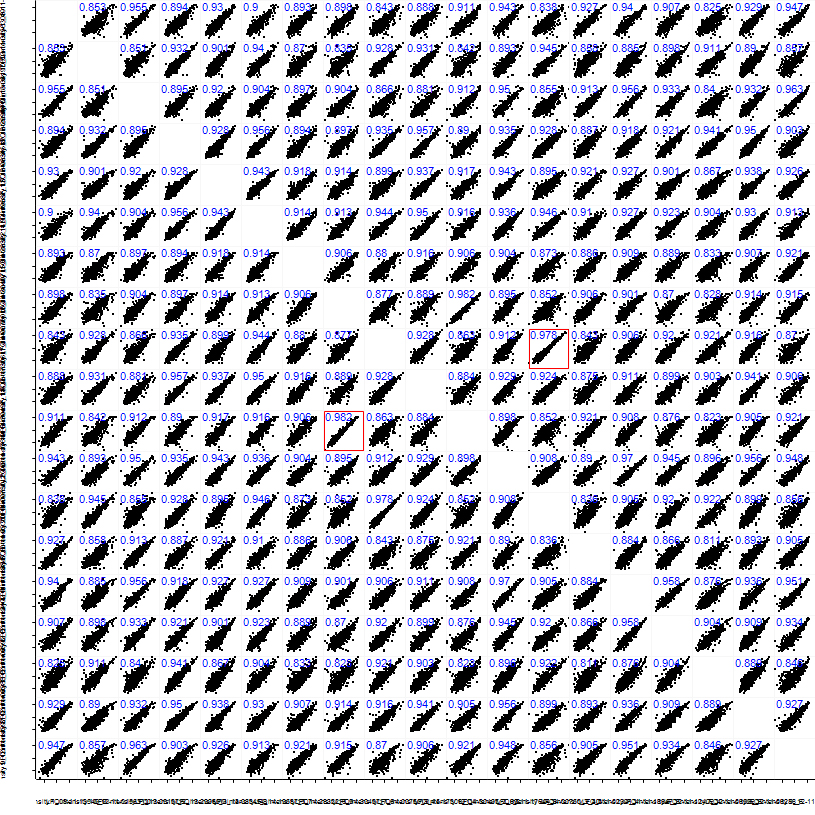

Supplement: Supplementary file 1 [file mmc1.zip › 161224_1_supp_556356_qcdrdv.jpg]

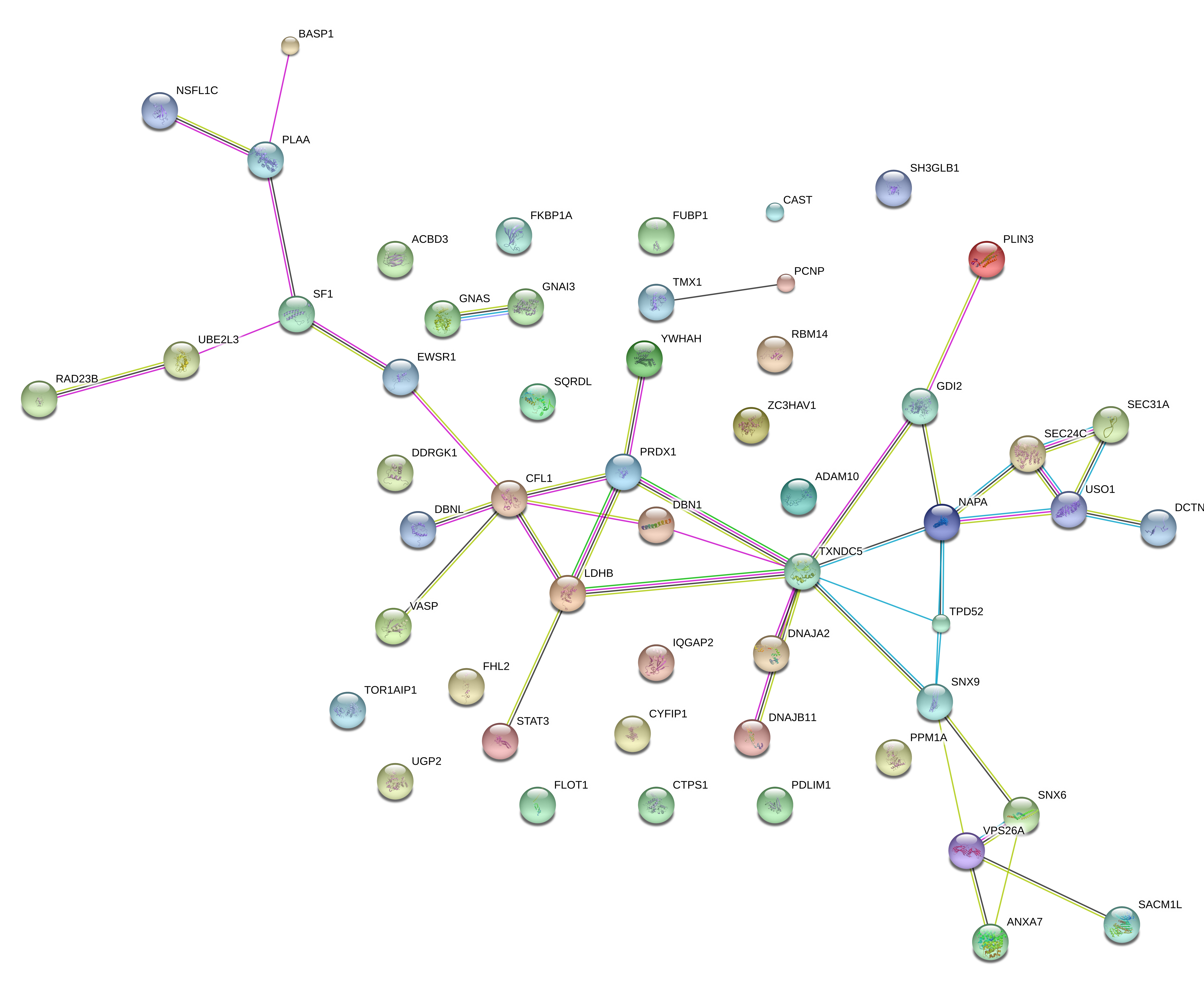

Supplement: Supplementary file 1 [file mmc1.zip › 161224_1_supp_556357_qc3r3v.jpg]

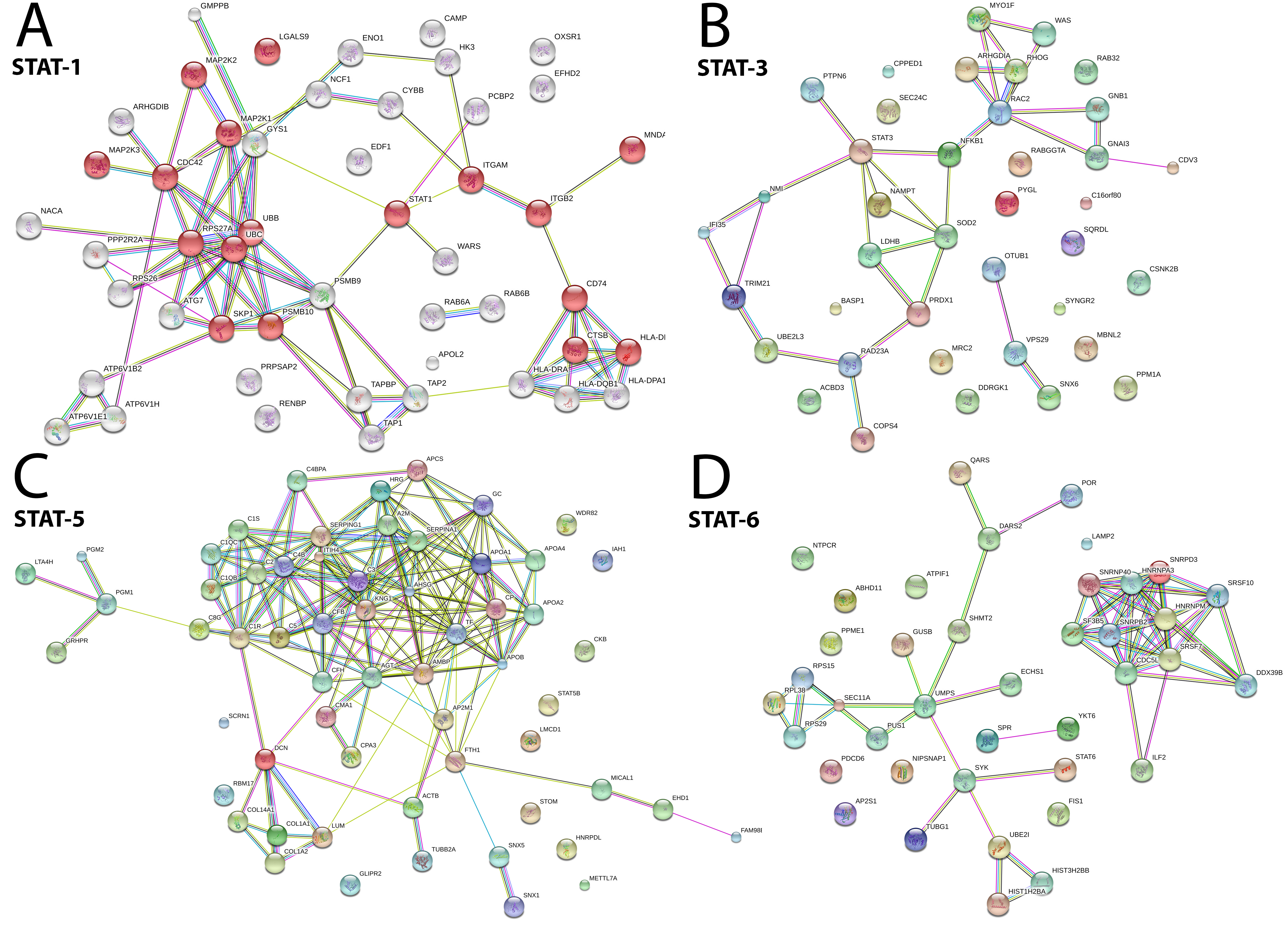

Supplement: Supplementary file 1 [file mmc1.zip › 161224_1_supp_556358_qc8r8v.jpg]

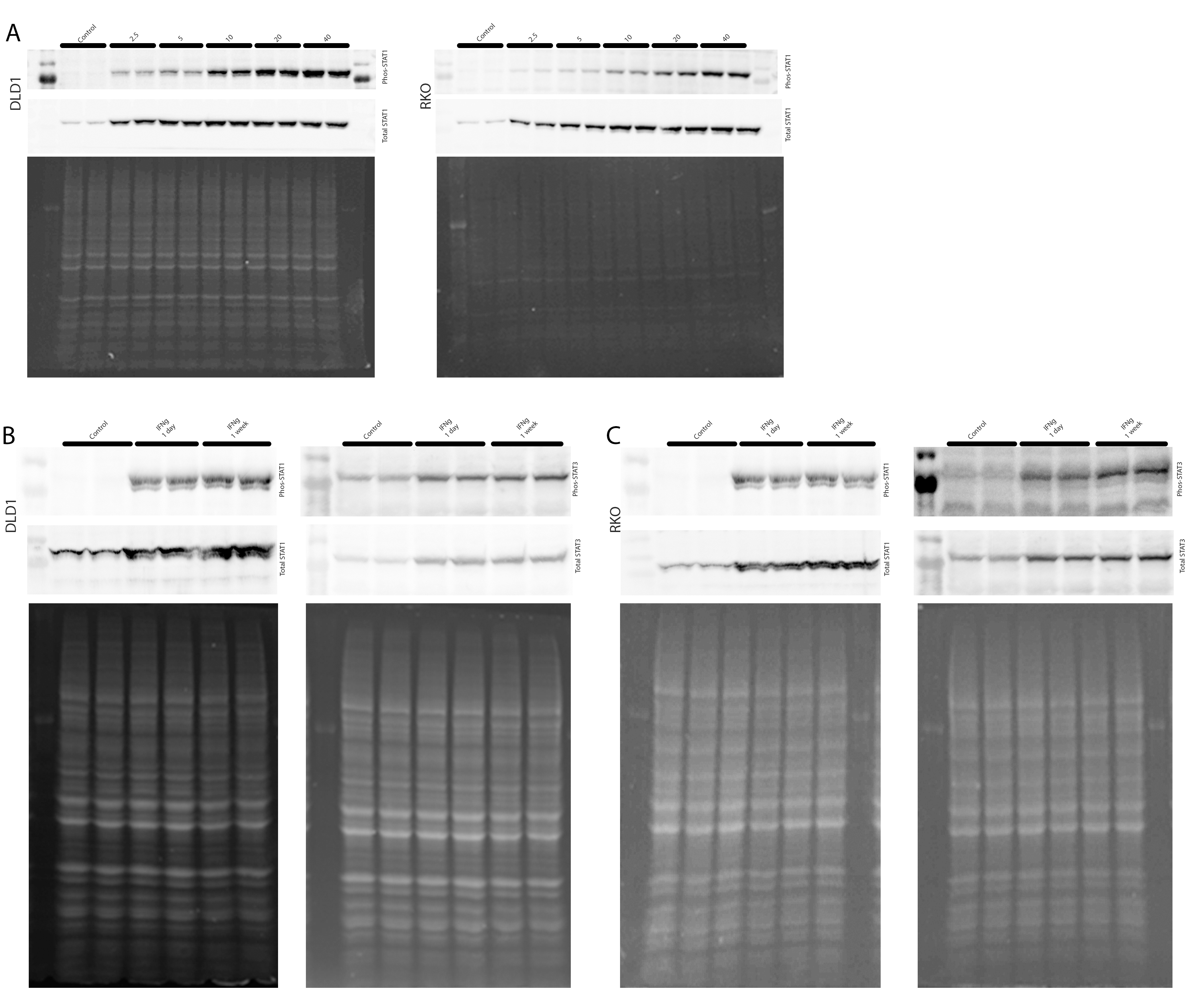

Supplement: Supplementary file 1 [file mmc1.zip › 161224_1_supp_556359_qcbrbv.jpg]
